# Psilocybin produces amplified acute responses and sex-specific long-term increases in sensorimotor gating in the mGlu5 knockout mouse model of schizophrenia

**DOI:** 10.1038/s41386-026-02501-3

**Published:** 2026-07-18

**Authors:** James J. Gattuso, Ahmed Kamal, Jennyfer M. Payet, Kato Havaux, Jenna Hendey, Bilgenur Bezcioglu, Zoe J. Phelan, Maddy Orchard, Divyesh Bhanot, Matthew W. Hale, Anthony J. Hannan, Thibault Renoir

**Affiliations:** 1 Florey Institute of Neuroscience and Mental Health, Parkville, VIC Australia; 2 Florey Department of Neuroscience and Mental Health, University of Melbourne, Parkville, VIC Australia; 3 Department of Anatomy and Physiology, University of Melbourne, Parkville, VIC Australia; 4 Department of Psychology, Counselling and Therapy, School of Psychology and Public Health, La Trobe University, Melbourne, VIC Australia; 5 Academic Quality Department, Melbourne Polytechnic, Melbourne, VIC Australia

**Keywords:** Schizophrenia, Schizophrenia, Genetics of the nervous system, Psychiatric disorders, Pharmacology

## Abstract

Psilocybin is a serotonergic psychedelic drug with emerging therapeutic applications, yet its actions under glutamatergic dysfunction, and relevance to schizophrenia and associated psychiatric disorders, remain unclear. We examined the acute and long-lasting effects of psilocybin (1 mg/kg, intraperitoneal) on behavioral and neural outcomes in the metabotropic glutamate receptor 5 (mGlu5) knockout (KO) mouse model of schizophrenia. The mGlu5 KO mice displayed psilocybin-induced hyperlocomotion, whereas wild-type (WT) mice did not. Additionally, male mGlu5 KO mice showed an amplified psilocybin-induced head-twitch response (HTR) compared with WT males, consistent with sex-dependent enhancement of 5-HT_2A_ receptor-mediated signaling. Acute psilocybin increased c-Fos expression in the claustrum of WT but not KO mice, suggesting intact mGlu5 signaling is required for psilocybin-evoked claustral recruitment. Psilocybin did not alter anxiety-like behavior in the light–dark box and increased immobility time in the Porsolt test. Strikingly, psilocybin produced a sustained normalization in prepulse inhibition (a measure of sensorimotor gating) in female KO mice, evident nine days after treatment. Together, these findings indicate that disrupted mGlu5 signaling amplifies acute responses to psilocybin and reveals a sex-dependent long-term effect on sensorimotor gating. These results refine understanding of glutamatergic–serotonergic interactions and motivate further work evaluating psilocybin across schizophrenia-relevant endophenotypes.

**Figure:**
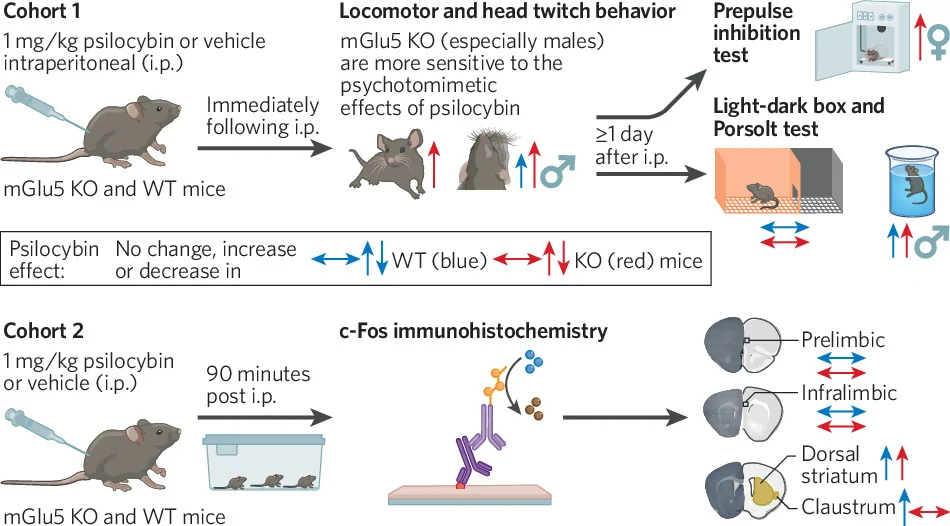

## Introduction

Schizophrenia is a severe, heterogeneous disorder characterized by positive, negative, and cognitive symptoms, and is frequently comorbid with anxiety [1, 2]. Although dopaminergic dysfunction has historically dominated explanatory models [3], glutamatergic and serotonergic disruptions are increasingly implicated [4, 5].

Metabotropic glutamate receptor 5 (mGlu5) modulates synaptic plasticity, learning, and emotional regulation [6–8]. mGlu5 knockout (KO) mice exhibit schizophrenia-relevant phenotypes, including hyperlocomotion, sensorimotor gating deficits, and social impairments [9–11], while first-line antipsychotics ameliorate hyperlocomotion and prepulse inhibition (PPI) deficits, supporting the model’s predictive validity [9, 12].

Psilocybin is dephosphorylated to psilocin, which binds multiple serotonin receptors, with high affinity for 5-HT_1A_ and 5-HT_2A_ receptors and lower-affinity interactions with D_2_/D_4_ receptors [13, 14]. In humans, psilocybin can induce psychosis-like alterations in perception and cognition, including perceptual distortions and an altered sense of self, largely linked to 5-HT_2A_ receptor activation [15–17]. Neuroimaging findings further suggest that psilocybin disrupts functional connectivity in ways that resemble early-stage psychosis, supporting its use as a model for probing cortical and thalamocortical dysfunction in schizophrenia [15].

A recent systematic review and meta-analysis [18] found the overall incidence of psychedelic-induced psychosis to be remarkably low—approximately 0.002% in population studies and 0.2–0.6% in uncontrolled and randomized clinical trials. Importantly, within the limited subgroup of historical uncontrolled trials that included patients with schizophrenia, the incidence of long-lasting psychotic symptoms was estimated at 3.8%, based on only two small non-randomized studies (*n* = 133) conducted more than fifty years ago. These early lysergic acid diethylamide (LSD) studies were characterized by a high risk of bias and outdated diagnostic criteria, limiting the reliability of their findings. Nonetheless, while the available evidence suggests that psychedelic-induced psychosis is rare overall, the risk may be heightened—but remains poorly quantified—in individuals with schizophrenia. Modern clinical trials continue to exclude participants with psychotic disorders or a family history of the disorder, leaving this important question unresolved [18]. This uncertainty provided the rationale for examining whether mGlu5 KO mice, as a schizophrenia-relevant model, exhibit altered acute sensitivity to psilocybin.

In rodents, psilocybin evokes hallucinogen-like behavior such as the head-twitch response (HTR), which is mediated by 5-HT_2A_ receptor activation [19]. 5-HT_2A_ receptor signaling is strongly implicated in the acute subjective effects of serotonergic psychedelics and is increasingly implicated in their therapeutic effects, although additional receptor systems and downstream mechanisms are likely to contribute [14, 16, 20, 21]. 5-HT_2A_ receptor signaling is also relevant to schizophrenia pathophysiology [22]. Individuals with schizophrenia exhibit altered 5-HT_2A_ receptor binding in the frontal cortex [23], and the 5-HT_2A_ receptor agonist 2,5-dimethoxy-4-methylamphetamine (DOM) exacerbates hyperlocomotion in mGlu5 KO mice relative to wild-type (WT) controls, suggesting enhanced behavioral sensitivity to 5-HT_2A_ receptor agonism under conditions of mGlu5 disruption [24]. Because 5-HT_2A_ receptors are highly expressed on cortical pyramidal neurons and modulate glutamatergic and dopaminergic neurotransmission, their interaction with mGlu5-dependent signaling may be particularly relevant to schizophrenia-related phenotypes [22].

Psilocybin has also been proposed to have potential therapeutic relevance for schizophrenia through plasticity-enhancing mechanisms that extend beyond its acute hallucinogen-like effects [25]. Candidate mechanisms include BDNF–TrkB signaling, downstream mTOR and AMPA receptor-associated signaling, synaptogenesis/spinogenesis, and dendritic spine remodeling [26–29]. Although these mechanisms were not directly assessed here, they provide a plausible framework through which psilocybin could produce delayed behavioral effects after the acute drug response has dissipated. This possibility is relevant because impaired plasticity has been reported in both patients with schizophrenia and mGlu5 KO mice [30–35].

We therefore characterized the acute and enduring effects of psilocybin in mGlu5 KO mice. Acutely, we tested whether psilocybin enhanced locomotor activity and the HTR in KO mice relative to WT controls, and mapped psilocybin-evoked c-Fos expression across cortico-striatal regions implicated in the HTR, locomotion, or psychedelic effects. These brain regions included the prelimbic and infralimbic cortex, claustrum, and dorsal striatum [36–38]. We also assessed longer-lasting effects on anxiety-like behavior, depressive-like behavior, and sensorimotor gating using the light-dark box (LDB), Porsolt, and prepulse inhibition (PPI) tests. We hypothesized that mGlu5 KO mice would show heightened acute behavioral responses and altered cortico-striatal activation, while psilocybin’s plasticity-related effects could produce delayed behavioral improvements (i.e., reduced anxiety-like and depression-like behavior and reduced sensorimotor deficits), which may be dissociable from the acute response.

## Methods and materials

### Animals

Male and female mGlu5 KO mice and WT littermates were generated from heterozygous × heterozygous breeding pairs on a C57BL/6J background and derived from a colony originally established at the Florey Institute of Neuroscience and Mental Health. Following weaning and genotyping, mice were group-housed (2–6 per cage) with same-sex conspecifics of the same genotype in individually ventilated cages to minimize potential confounding effects of genotype-dependent social interactions on behavioral outcomes, consistent with our previous work [39–41]. Mice had ad libitum access to standard chow and drinking water, in the 12:12 light/dark cycle (lights were on from 7 a.m. to 7 p.m.). Mice were approximately 4 months old at the time the experiments were conducted. For behavioral experiments, a total of 96 mice were used (Supplementary Table 1). For c-Fos analysis, a separate cohort of 61 behaviorally naive mice was used (Supplementary Table 2). All animal experiments were approved by the Florey Institute Animal Ethics Committee, following the guidelines of the National Health and Medical Research Council (NHMRC).

### Drugs

Psilocybin (1 mg/kg), provided by Usona Institute Inc., was dissolved in 0.9% saline solution, and administered via intraperitoneal (i.p.) injection. This dose was based on previous research [39, 41, 42] where it yielded hallucinogenic-like properties (by eliciting a significant number of head twitches) and exhibited therapeutic behavioral effects as well as increasing dendritic growth in the prefrontal cortex [26]. Control mice received vehicle treatment under the same conditions (volume of injection 10 ml/kg, 0.9% saline, i.p.). Mice were injected with a single dose of psilocybin 1 mg/kg, i.p. A subset of mice (*n* = 47) received a second injection of psilocybin (1 mg/kg, i.p.) or saline two weeks after the initial administration. Locomotor activity and HTR were recorded again to assess whether behavioral responses to psilocybin differed between the first and second administrations.

### Behavioral experiments

All behavioral testing was conducted during the light cycle (9 a.m. - 5 p.m.). Treatment groups were scheduled in a counterbalanced manner to reduce possible circadian or time-of-day effects, ensuring that mice receiving psilocybin and those given saline were evenly tested in morning and afternoon periods. In all experiments, mice were acclimatized to the behavioral room for at least 30 min before testing. Treatment groups were pseudo-randomized using baseline LDB performance collected one week before dosing to balance pre-treatment avoidance/exploratory behavior. LDB was used for stratification because these data had been processed at the time of allocation and provided a relevant baseline measure of behavior in an aversive context. Baseline PPI and Porsolt test data were also collected before treatment but had not yet been processed and were therefore unavailable for stratification; these baseline measures are presented in Supplementary Fig. 5 and did not differ between mice subsequently assigned to saline or psilocybin treatment groups.

**Fig. 1:**
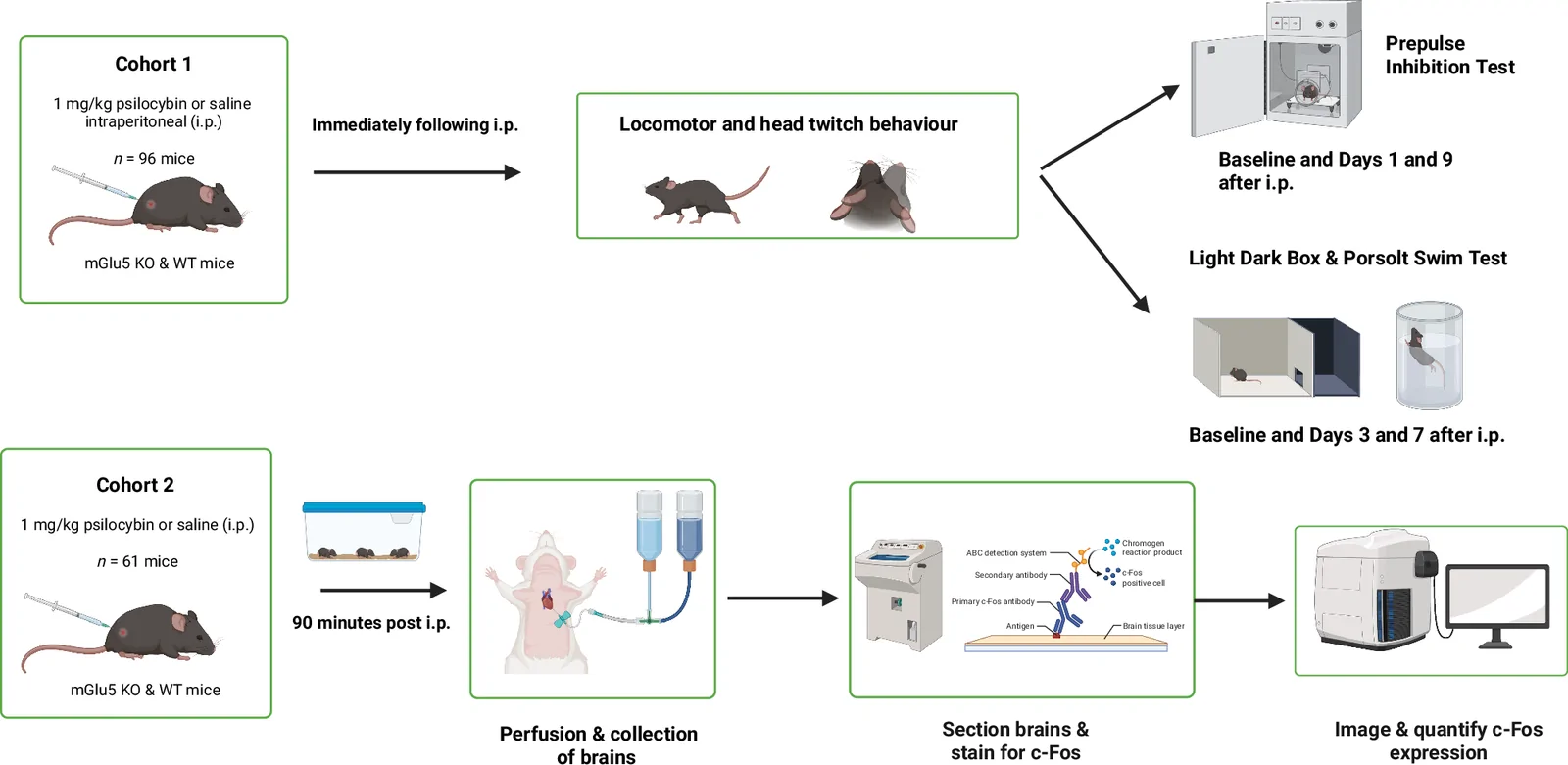
Experimental design and timeline. Cohort 1 (*n* = 96; mGlu5 knockout and wild-type mice) received psilocybin (1 mg/kg, i.p.) or saline and underwent acute behavioral assays immediately following treatment. Acute measures included locomotor activity and head-twitch response, followed by post-acute behavioral assessments of sensorimotor gating (prepulse inhibition) on days 1 and 9, and anxiety- and depression-like behaviors in the light–dark box and Porsolt test on days 3 and 7 after treatment. All post-acute behavioral tests were also conducted at baseline (3–7 days before drug administration). Cohort 2 (*n* = 61; mGlu5 knockout and wild-type mice) received the same treatments and were perfused 90 min post-injection for c-Fos immunohistochemistry to investigate the neural activation patterns underlying the acute effects of psilocybin administration. Brains were sectioned, stained, and quantified for c-Fos expression. Although not shown in the schematic, a subset of animals (*n* = 47) from cohort 1 received a second dose of psilocybin or saline 2 weeks after the first dose where locomotor and head-twitch behaviors were reassessed.

Post-acute behavioral timepoints were selected to examine both early and more sustained effects of psilocybin after resolution of the acute drug response (psilocin has a plasma clearance of 4 h in mice [43]). PPI was assessed on days 1 and 9, whereas the LDB and Porsolt tests were conducted on days 3 and 7. Within this sequence, day 3 represented the earliest practical post-acute timepoint following the day 1 PPI assessment and an intervening recovery day, while day 7 provided a later timepoint to assess whether behavioral effects persisted across the first week or had begun to wane. The spacing of behavioral test days allowed at least one full recovery day between assays, thereby reducing the likelihood of carryover effects from prior testing. These timepoints were also informed by our previous work showing that psilocybin reduced grooming in male SAPAP3 KO mice at 3 and 8 days, but not 1 day, after injection [41]. Repeated assessment across post-acute timepoints in the same cohort also increased statistical efficiency while minimizing animal use. A schematic of the experimental timeline is shown in Fig. 1. Individual mouse identifiers, cohort, sex, genotype, and treatment allocation are provided in Supplementary Table 3.

### Locomotor activity

Locomotor activity was measured for 30 min after acute psilocybin administration by placing mice in a 27.3 cm × 27.3 cm × 20.3 cm photo-beam activity chamber (Med Associates Inc., VT, USA).

### Head-twitch response (HTR)

In the 30 min immediately after injection, mice were placed in the locomotor cell and were video recorded for the first 15 min following drug administration from a top view (the video data was captured at a resolution of 1920 × 1088 pixels with a frame rate of 25 frames per second). The behavior was manually scored by a blinded and trained researcher. The HTR was measured while mice were completing their locomotor behavior. This behavior was conducted in alignment with previous methodologies [41].

### Light-dark box (LDB)

This test was conducted in alignment with well-established protocols from our laboratory [41]. The LDB test was conducted using square photo-beam arenas (27.3 ×  27.3 × 20.3 cm; Med Associates Inc., VT, USA) that were partitioned into illuminated and dark sections using a black Plexiglas divider. The illuminated side was maintained at a brightness of 750 ± 10 lux. Mice were placed in the dark compartment via an opening in the top of the insert and allowed to freely explore both compartments for 10 min. Anxiety-like behavior was evaluated based on the proportion of time spent in the brightly lit compartment. Additional parameters such as the total distance moved, the latency to the light compartment and the number of transitions between the two compartments were also recorded [44].

### Porsolt test

The Porsolt test was employed to evaluate antidepressant-like effects, following previously established protocols [39]. In brief, mice were individually placed into 2-L glass beakers containing 1.5 L of water maintained at 25 ± 2 °C, with no possibility of escape, and observed for a total duration of 6 min. To minimize potential olfactory confounds, males and females were tested in separate beakers. The primary variable of interest was the duration of immobility, defined as floating with minimal movement to stay afloat, which is considered indicative of a passive coping response to stress and is sensitive to antidepressant treatment [45]. Immobility was quantified during the final 4 min of testing using an automated tracking system (Depression Scan, Clever Sys Inc., Reston, VA). Additionally, in alignment with previous methodologies we conducted a baseline session, 5 days prior to drug administration [46]. Prior to testing, mice were acclimated to the testing room for 30 min immediately following their LDB session.

### Prepulse inhibition (PPI)

Prepulse inhibition (PPI) of acoustic startle was employed to evaluate acoustic startle reactivity and sensorimotor gating, using an automated SR-LAB system (San Diego Instruments, USA), in accordance with previously published procedures [47]. A more detailed explanation of the PPI test is found in the supplementary file.

### c-Fos immunohistochemistry and quantification

Ninety minutes after psilocybin or saline injection, mice were perfused with PBS followed by 4% PFA, and brains were post-fixed, cryoprotected, and coronally sectioned (30 µm). Free-floating sections underwent c-Fos immunohistochemistry adapted from prior published protocols [48], with avidin-biotin complex (ABC)-based chromogen detection.  Whole-slide images were acquired on a Zeiss Axioscan 7 and c-Fos-positive nuclei were quantified in QuPath using standardized ROIs and detection parameters with manual verification. Full methodological details, ROI rationale, and representative images are provided in the Supplementary Methods and Supplementary Figs. 1–4.

### Statistical analysis

Statistical analyses were conducted in GraphPad Prism and R, with significance set at *p* < 0.05 and statistical trends defined as 0.05 ≤ *p* ≤ 0.06. Acute behavioral data were analyzed using three-way ANOVAs or linear mixed-effects models (for repeated measures). HTR effects were further examined using sex-stratified difference-in-differences analyses, and associations between behavioral measures were assessed using correlation and regression models incorporating genotype interactions where applicable. Given the small and unequal c-Fos group sizes (*n* = 5–10 mice per group), assumptions of normality and homogeneity of variance were carefully evaluated, as conventional ANOVA may be less robust to assumption violations in small, unbalanced designs. For post-acute primary behavioral outcomes, linear mixed-effects models included genotype, treatment, sex, time, and their relevant interactions as fixed effects, with mouse ID included as a random intercept to account for repeated measures. Post-hoc comparisons were adjusted for multiple comparisons, where applicable, using Šidák or Holm–Bonferroni correction, as appropriate. Full statistical procedures are provided in the Supplementary Methods.

## Results

### mGlu5 knockout mice exhibit enhanced sensitivity to psilocybin’s acute behavioral effects

**Fig. 2:**
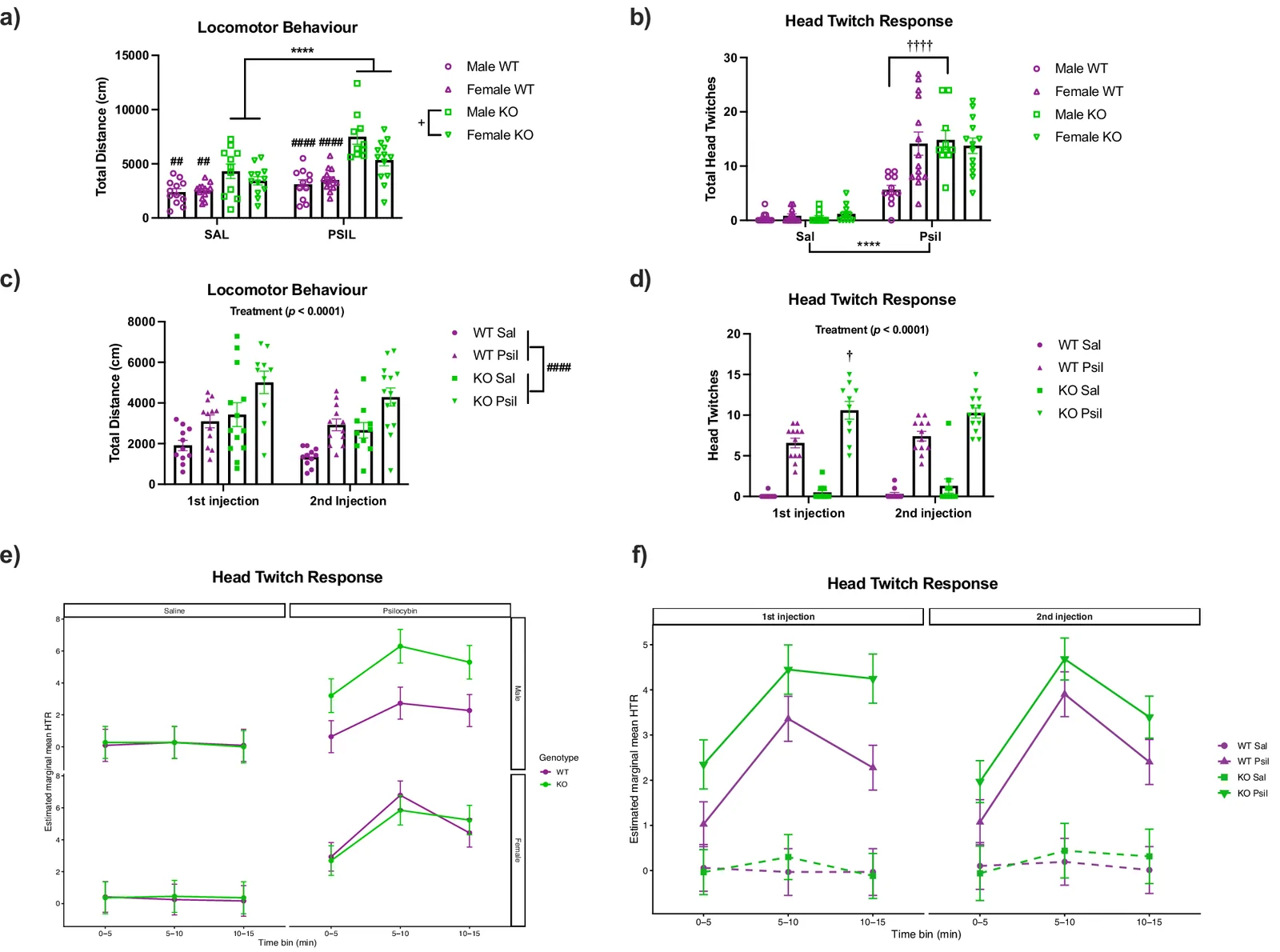
The effects of mGlu5 deletion and psilocybin treatment on locomotor and head-twitch behaviors. **a** The locomotor test was conducted for 30 min immediately following psilocybin administration. Psilocybin induced hyperlocomotion in mGlu5 KO (*p* < 0.0001) but not WT mice (*p* = 0.103). **b** HTR was measured for 15 min immediately following psilocybin administration. Psilocybin produced a significantly greater HTR in male mGlu5 KO than WT mice (*p* < 0.0001). **c** A second dose of psilocybin produced similar hyperlocomotion effects compared to the first dose. **d** A second dose of psilocybin produced a comparable number of head twitches compared to the first dose. KO mice that received psilocybin had a greater number of head twitches compared to WT mice receiving psilocybin and this effect was most robust at the 1^st^ injection. **e**, **f** Temporal profile of psilocybin-induced HTR. **e** Psilocybin’s peak effect occurred at 5–10 min post administration (*p* < 0.0001) and was not influenced by genotype (*p* = 0.413). **f** The temporal profile of psilocybin-induced HTR did not differ between the first and second injection and prior psilocybin exposure did not alter subsequent psilocybin-induced HTR. *n* = 10–14 mice per group. Panels a–d display means ± SEM, whereas panels **e**, **f** show model-derived estimated marginal means ± 95% confidence intervals. WT wild-type, KO knockou, Sal saline, Psil psilocybin 1 mg/kg. ****p < 0.0001, Psil vs Sal, ^##^*p* < 0.01, ^####^*p* < 0.0001, WT vs KO; ^+^*p* < 0.05, KO male vs KO female. ^†^*p* < 0.05, [(KO − WT)_psil_,_1st injection_ − (KO − WT)_sal_,_1st injection_]; ^††††^*p* < 0.0001, [(KO − WT)_Psil, Male_ − (KO − WT)_Sal, Male_]. For panels **c** and **d**, the bold text above the grouped bars represents a significant main effect of treatment.

For locomotor activity, a three-way ANOVA was performed (Fig. 2a). We found a significant genotype × treatment interaction (*F*_1,88_ = 7.22, *p* = 0.008, *η*^2^ = 0.039), and post-hoc analysis found that psilocybin increased locomotion in mGlu5 KO (*p* < 0.0001) but not WT mice (*p* = 0.103). KO mice exhibited greater locomotor activity when receiving saline or psilocybin compared to WT mice (*p* = 0.006 and *p* < 0.0001, respectively). Additionally, a sex × genotype interaction (*F*_1,88_ = 8.13, *p* = 0.005, *η*^2^ = 0.042) was observed and post-hoc analysis found that male mGlu5 KO mice had greater locomotion than female KO mice overall (*p* = 0.018). No other significant interactions were revealed.

We conducted a three-way ANOVA to assess the hallucinogenic-like properties of psilocybin (1 mg/kg, i.p.) (via measuring the HTR) in male and female WT and mGlu5 KO mice (Fig. 2b). A main effect of psilocybin (F_1,85_ = 160, *p* < 0.0001, *η*^2^ = 0.563) shows that psilocybin significantly increased the HTR compared to saline. Interestingly, we found a significant treatment × genotype × sex interaction (F_1,85_ = 7.95, *p* = 0.008, *η*² = 0.026). Difference-in-differences analysis revealed that psilocybin-treated male mGlu5 KO mice exhibited a markedly greater HTR than psilocybin-treated male WT mice (*p* < 0.0001), whereas psilocybin-induced HTR did not differ significantly between female KO and WT mice (*p* = 0.793) (Fig. 2b).

A subset of mice received a second injection of psilocybin (1 mg/kg, i.p.) or saline  two weeks after their first treatment session (Fig. 2c, d). As no sex or sex × treatment effects were observed (*p* ≥ 0.076), sexes were combined. For locomotion, there was no treatment × injection number interaction (F_1,39_ = 0.194, *p* = 0.662), but KO mice showed greater locomotion than WT (F_1,46_ = 25.7, *p* < 0.0001) and psilocybin increased locomotion (F_1,46_ = 25.0, *p* < 0.0001). For HTR, there was no treatment × injection number interaction (F_1,84_ = 0.0732, *p* = 0.787), but psilocybin increased HTR (F_1,84_ = 366, *p* < 0.0001) with a genotype × treatment interaction (F_1,84_ = 10.2, *p* = 0.002). Difference-in-differences analyses showed greater psilocybin-induced HTR in KO vs WT at the first injection (*p* = 0.010). At the second injection, this effect emerged in a sensitivity analysis following removal of an outlier (*p* = 0.006).

HTR temporal dynamics showed a significant treatment × time interaction (F_2,186_ = 50.3, *p* < 0.0001) and there was no treatment  × genotype interaction (F_2,186_ = 0.894, *p* = 0.413), with peak HTR occurring at 5–10 min (*p* < 0.0001) (Fig. 2e). This profile was unchanged by injection number (F_2,180_ = 1.85, *p* = 0.160) or prior psilocybin exposure (time interaction: F_2,70_ = 0.680, *p* = 0.512; main effect: F_1,16.3_ = 0.0795, *p* = 0.781), indicating preserved temporal dynamics and magnitude following repeated administration (Fig. 2f).

### Psilocybin increases c-Fos activity in the claustrum of WT but not KO mice

**Fig. 3:**
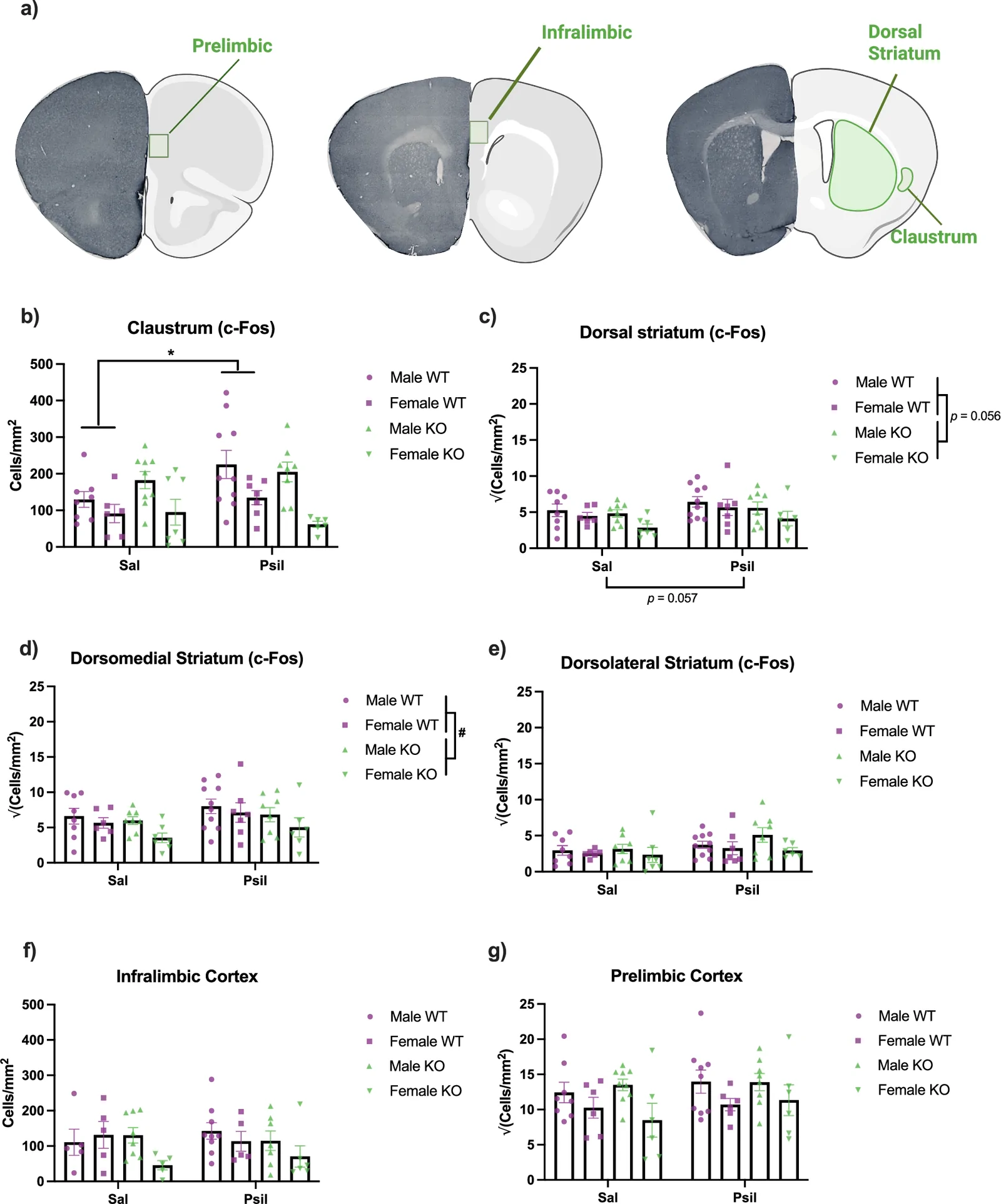
c-Fos expression following psilocybin administration across brain regions of interest. **a** Representative coronal sections showing regions of interest: prelimbic cortex, infralimbic cortex, and dorsal striatum with the claustrum outlined. **b** In the claustrum, a genotype × treatment interaction was observed (*p* = 0.046), with WT mice treated with psilocybin showing higher c-Fos density compared to WT mice treated with saline (*p* = 0.044). **c** In the dorsal striatum, males exhibited higher c-Fos density than females (*p* = 0.032), with trend-level main effects of genotype (*p* = 0.056) and treatment (*p* = 0.057). **d** In the dorsomedial striatum, we found that mGlu5 KO mice had less c-Fos than WT mice, (*p* = 0.045), and males had higher c-Fos density than females (*p* = 0.043). Psilocybin did not significantly alter c-Fos compared to saline (*p* = 0.081). **e** There were no significant effects in the dorsolateral striatum (*p* ≥ 0.062). **f** In the infralimbic cortex, no significant main effects or interactions were detected (*p* ≥ 0.092 for all effects). **g** In the prelimbic cortex, males had higher c-Fos density than females (*p* = 0.004). *n* = 5–10 mice per group. Bars represent mean ± SEM; individual data points represent mice. WT wild-type, KO knockout, Sal saline, Psil psilocybin 1 mg/kg. *p < 0.05, WT Psil vs WT Sal.

We first examined c-Fos density in the claustrum. Residuals violated homoscedasticity (*ρ* = 0.37, *p* = 0.002), so we conducted a Welch-type ANOVA. This revealed a significant main effect of sex (*p* < 0.0001), with males showing higher c-Fos density than females. There was also a significant genotype × treatment interaction (*p* = 0.046). Post-hoc Welch *t*-tests showed that WT mice treated with psilocybin had greater c-Fos density than WT mice treated with saline (*p* = 0.044; Fig. 3b), whereas no difference was observed between KO mice treated with saline and those treated with psilocybin (*p* > 0.999). No other interactions were significant.

In the dorsal striatum, residuals were not normally distributed (Shapiro–Wilk, *p* = 0.001) and the data violated homoscedasticity (*ρ* = 0.51, *p* < 0.0001). A square-root transformation normalized residuals (Shapiro-Wilk, *p* = 0.643) but heteroscedasticity remained (*ρ* = 0.32, *p* = 0.006). Therefore, we used a Welch-type ANOVA, which revealed a significant main effect of sex (*p* = 0.032), with males again showing higher c-Fos density when compared to females. There were trend-level main effects of genotype (*p* = 0.056) and treatment (*p* = 0.057), with KO mice showing numerically lower c-Fos density than WT mice, and psilocybin-treated mice showing numerically higher density than saline-treated mice (Fig. 3c). However, because the dorsal striatum can be further subdivided into the functionally distinct dorsomedial striatum (DMS) and dorsolateral striatum (DLS) [49], we conducted additional analyses of these subregions (Fig. 3d, e). For the DMS, the data violated assumptions of normality (Shapiro-Wilk *p* = 0.008) and homoscedasticity (*ρ* = 0.483, *p* < 0.0001), however, these were both corrected after a square-root transformation. Thus, we used a three-way ANOVA, and found a significant effect of genotype (F_1,52_ = 4.23; *p* = 0.045, *η*^2^ = 0.065) and sex (F_1,52_ = 4.33; *p* = 0.043, *η*^2^ = 0.064), with mGlu5 KO mice having less c-Fos than WT mice, and males having more c-Fos than females. There was no significant effect of psilocybin (F_1,52_ = 3.16; *p* = 0.081, *η*^2^ = 0.048) or any significant interactions. For the DLS, likewise a square-root transformation was applied as the data also did not meet assumptions, however, these assumptions remained violated after the transformation. Thus, we conducted a permutation ANOVA and found that there was no significant effect of sex (F_1,52_ = 3.38, *p* = 0.072), treatment (F_1,52_ = 3.63, *p* = 0.062), or genotype (F_1,52_ = 0.255, *p* = 0.613), or any significant interactions.

For the infralimbic cortex (Fig. 3f), a three-way ANOVA showed no significant main effects of sex (*F*_1,42_ = 2.97, *p* = 0.092, *η*^2^ = 0.058); genotype (*F*_1,42_ = 2.91, *p* = 0.095, *η*^2^ = 0.056); or treatment (*F*_1,42_ = 0.09, *p* = 0.769, *η*^2^ = 0.002) and no significant interactions. In the prelimbic cortex, residuals violated normality (Shapiro–Wilk, *p* = 0.003) but not homoscedasticity (*ρ* = 0.11, *p* = 0.212). A square-root transformation did not resolve the violation of normality (Shapiro–Wilk, *p* = 0.017), so we conducted a permutation ANOVA. This revealed a significant main effect of sex (*p* = 0.004), with males again showing higher c-Fos density compared to females (Fig. 3g). There were no significant effects of genotype (*p* = 0.978) or treatment (*p* = 0.246), and no significant interactions.

### Psilocybin does not alter anxiety-like behavior but increases immobility time in the Porsolt test

**Fig. 4:**
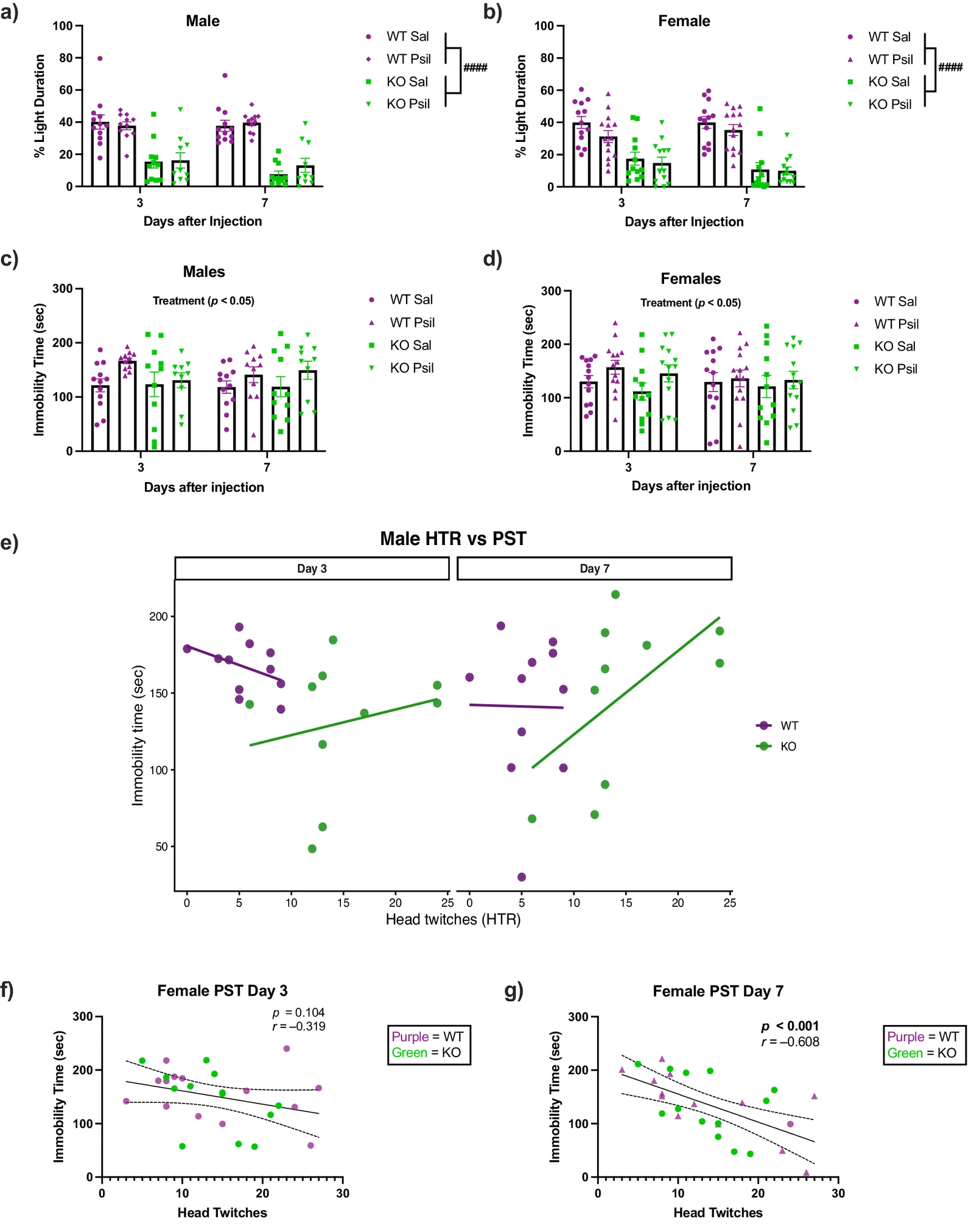
The effects of mGlu5 deletion and psilocybin on behaviors relevant to anxiety and depression. **a**,**b **The LDB test was performed in male and female mice, 3 and 7 days after drug administration. KO mice had reduced light duration compared to WT mice (*p* < 0.0001). Psilocybin did not alter light duration in WT or KO mice (*p* = 0.544). **c**, **d** The PST was conducted in male and female mice 3 and 7 days after drug administration. No genotype effect was present (*p* = 0.353). However, psilocybin significantly increased immobility time (*p* = 0.011). **e** The association between the number of psilocybin-induced head twitches and immobility time in male mice 3 and 7 days after administration stratified by genotype. There was no significant association in either group (*p* ≥ 0.118). **f**, **g** The association between the number of psilocybin-induced head twitches and immobility time in female mice 3 and 7 days after administration. At the day 7 time point in females, mice that exhibited more head twitches tended to have reduced immobility time (*p* < 0.001). **a–d** *n* = 10–14 mice per group. **e** 10–11 mice per group. **f**, **g** *n* = 13–14 mice per group. **e–g** Displays only mice that received psilocybin. Bars represent the mean ± SEM; individual data points represent mice. WT wild-type, KO knockout, Sal saline, Psil psilocybin 1 mg/kg. ^####^*p* < 0.0001, WT vs KO collapsed across sex. For panels **c** and **d**, the bold text above the grouped bars represents a significant main effect of treatment, collapsed across sex.

A linear mixed-effects model was conducted to analyze the effects of psilocybin treatment on anxiety-like behavior in the LDB (Fig. 4a, b). There was no significant effect of sex (F_1,96.0_ = 0.219, *p* = 0.641), treatment (F_1,96.0_ = 0.371, *p* = 0.544) or a sex × treatment interaction (F_1,96.0_ = 1.66, *p* = 0.200), however, there was a significant genotype × time interaction (F_1, 96_ = 9.66, *p* = 0.002). mGlu5 KO mice had reduced light duration compared to WT mice at both day 3 and day 7 (*p* < 0.0001 respectively) and KO mice had reduced light duration at day 7 compared to day 3 (*p* < 0.001), whereas there was no significant difference between time points for WT mice (*p* = 0.541). For the subsidiary measures, mGlu5 KO mice traveled significantly less than WT mice, had increased latency to the light side and had fewer transitions to the light side (Supplementary Fig. 6). However, psilocybin did not alter any subsidiary measures such as latency to the light, distance traveled, or number of transitions in male or female mice (Supplementary Fig. 6).

In the Porsolt test, we found a main effect of treatment, whereby mice receiving psilocybin had greater immobility time compared to mice receiving saline (F_1,96_ = 6.73, *p* = 0.011). There was no significant effect of sex (F_1,96_ = 0.00770, *p* = 0.930) and genotype (F_1, 96_ = 0.872, *p* = 0.353) or time (F_1,96_ = 0.709, *p* = 0.401). There were no significant interactions. Female but not male mGlu5 KO mice had significantly greater latency to float compared to WT mice and psilocybin did not significantly alter the latency to float in male or female mice (Supplementary Fig. 6).

Following up on the significant treatment effect revealed in the Porsolt test, we further investigated the potential association with acute 5-HT_2A_ receptor activation by performing correlation analyses between the HTR and immobility time. In the males, as WT and KO mice had significantly different psilocybin-induced HTR, genotype was accounted for statistically. We found no evidence that the relationship between HTR and immobility differed by genotype at either day 3 or day 7 (HTR × genotype interaction: day 3, *p* = 0.345; day 7, *p* = 0.381). After controlling for genotype, HTR was not significantly associated with immobility time at day 3 (partial *r* = 0.105, *p* = 0.660) or day 7 (partial *r* = 0.361, *p* = 0.118) (Fig. 4e). For females, we found a significant negative correlation between psilocybin-induced HTR and immobility time at day 7 (*p* < 0.001, *r* = −0.608) but not day 3 (*p* = 0.104, *r* = −0.319) (Fig. 4f, g).

### Psilocybin produced long-term restoration of PPI in female mGlu5 KO mice

**Fig. 5:**
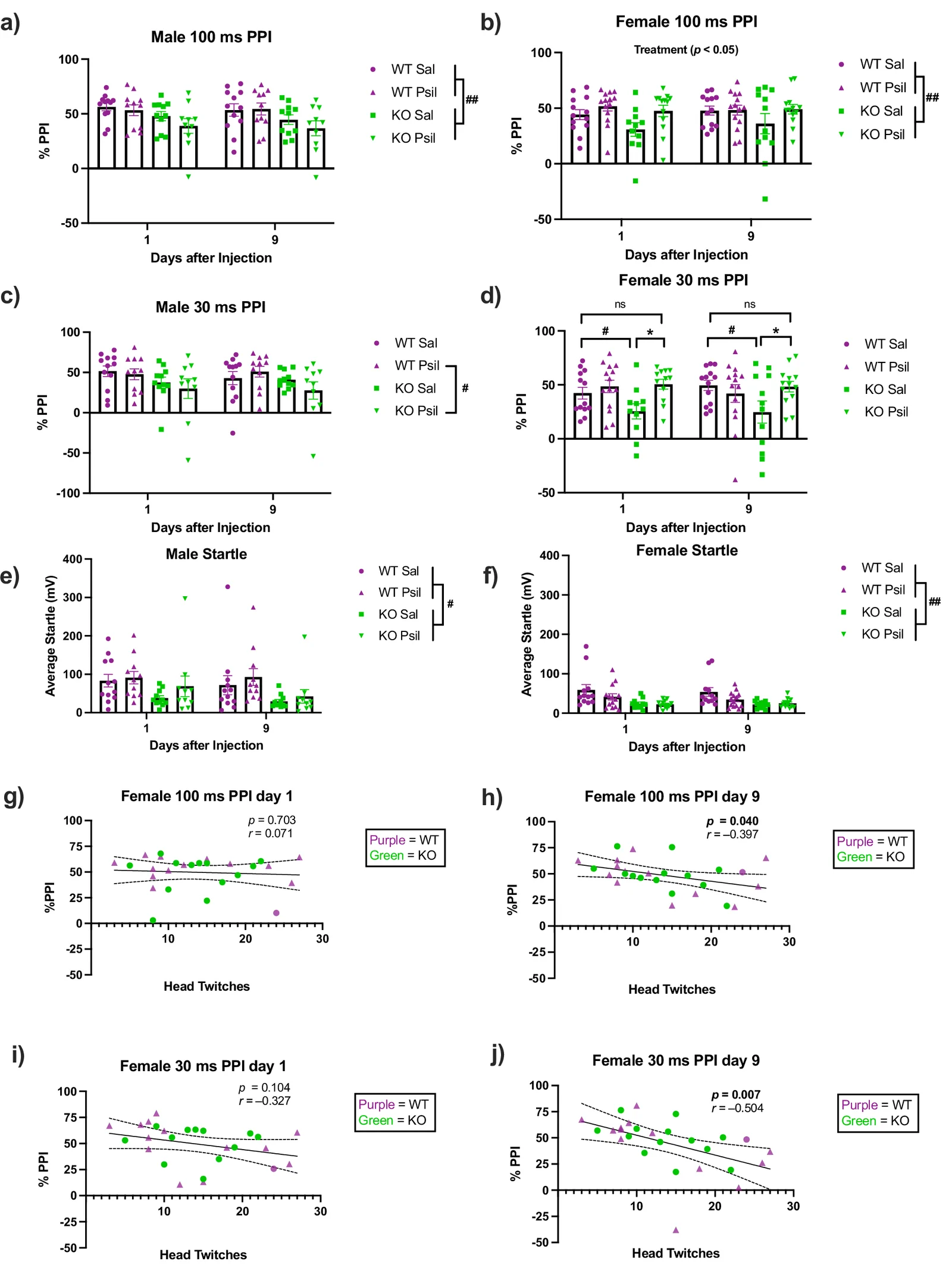
The effects of psilocybin and mGlu5 deletion on prepulse inhibition behavior. **a**, **b** mGlu5 KO mice had significantly lower PPI compared to WT mice (*p* = 0.001). For 100 ms PPI, psilocybin increased PPI in female (*p* = 0.013) but not male mice (*p* = 0.288). **c**, **d** For 30 ms PPI in males, psilocybin did not alter PPI performance. For 30 ms PPI in females, KO mice treated with saline had significantly lower PPI compared to WT mice treated with saline (*p* = 0.018), and psilocybin administration increased PPI in KO mice compared to saline-treated KO mice (*p* = 0.020). There was no significant difference between WT mice treated with saline and KO mice treated with psilocybin (*p* > 0.999). **e** Male KO mice had reduced startle response compared to WT mice (*p* = 0.022), and there was no effect of time (*p* = 0.152) or treatment (*p* = 0.286), or any significant interactions. **f** Similar findings occurred for the female startle, whereby, female KO mice had reduced startle compared to WT mice (*p* = 0.002) and there was no effect of treatment (*p* = 0.265) or time (*p* = 0.208) or any significant interactions. **g–j** Psilocybin-induced HTR plotted against PPI performance in female mice. **a–f** *n* = 10–14, **g–j** *n* = 13–14 mice per group. Bars represent mean ± SEM; individual data points represent mice. WT wild-type, KO knockout, Sal saline Psil psilocybin 1 mg/kg. ^#^*p* < 0.05, ^##^*p* < 0.01, *p < 0.05, KO Sal vs KO Psil. ns, was a non-significant comparison between KO Psil vs WT Sal. For panels **a** and **b**, ^##^*p* < 0.01 represents a main effect of genotype collapsed across sex.

To assess the impact of psilocybin on sensorimotor gating, PPI was measured in WT and mGlu5 KO mice using 100 ms and 30 ms interstimulus intervals (ISI) across two time points (1- and 9-days post-treatment; Fig. 5a–d). For 100 ms PPI, there was a main effect of genotype (F_1,94.1_ = 11.5, *p* = 0.001), with mGlu5 KO mice having significantly lower PPI compared to WT mice. There was no significant effect of time (F_1,93.8_ = 0.135, *p* = 0.714), however, there was a significant treatment × sex interaction (F_1,94.1_ = 6.32, *p* = 0.014), where post hoc analysis revealed that psilocybin significantly increased PPI compared to saline in females (*p* = 0.013) but not males (*p* = 0.288). There were no other significant interactions.

For 30 ms PPI, we found no significant effect of time (F_1,94.4_ = 0.05, *p* = 0.821) and a significant treatment × sex × genotype interaction (F_1,94.8_ = 4.25, *p* = 0.042), where post-hoc analysis revealed that female mGlu5 KO mice that received psilocybin had significantly greater PPI compared to female KO mice that received  saline (*p* = 0.020). Additionally, female KO mice that received saline, had significantly lower PPI compared to female WT mice that received  saline (*p* = 0.018) and KO mice that received psilocybin were not significantly different to WT mice that received saline (*p* > 0.999) suggesting normalization of PPI response. For male mGlu5 KO mice, psilocybin did not alter PPI compared to saline (*p* = 0.250) and KO mice treated with psilocybin had lower PPI compared to WT mice treated with psilocybin (*p* = 0.014). There was no significant PPI difference between male WT and KO mice treated with  saline (*p* = 0.353).

As male and female mGlu5 KO mice exhibited reduced pulse-alone startle amplitude compared to WT mice (F_1,40_ = 5.66, *p* = 0.022 and F_1,49_ = 10.99, *p* = 0.002 respectively) (Fig. 5e, f), we conducted a sensitivity analysis in which pulse-alone startle amplitude was included as a covariate to assess whether the PPI findings were robust to baseline differences in startle reactivity. The covariate-adjusted analysis yielded a similar pattern of results to the primary analysis (Supplementary Fig. 7).

To determine the potential role of the hallucinogenic-like experience in the enduring effects of psilocybin on sensorimotor gating, we performed correlational analyses between the HTR and PPI in psilocybin-treated mice. At 9 days post-injection, female mice that tended to have a greater number of psilocybin-induced HTR, had lower PPI (*p* = 0.007, *r* = –0.504) (Fig. 5j). However, there was no significant correlation at 1 day post injection (*p* = 0.703, *r* = 0.071) (Fig. 5g). For males, there was no evidence that the relationship between HTR and PPI differed by genotype at either prepulse interval on day 1 or day 9 (HTR × genotype interaction: all *p* ≥ 0.287). After controlling for genotype, HTR was not significantly associated with PPI in male mice at day 1 (30 ms: partial *r* = –0.033, *p* = 0.889; 100 ms: partial *r* = –0.015, *p* = 0.951) or day 9 (30 ms: partial *r* = –0.147, *p* = 0.537; 100 ms: partial *r* = -0.192, *p* = 0.419) (Supplementary Fig. 8). Together, these findings indicate that, unlike in females, psilocybin-induced HTR was not significantly associated with subsequent PPI in males.

As expected, for both 100 ms and 30 ms PPI, as the prepulse intensity increased so did the PPI (Supplementary Fig. 9).

## Discussion

### Validating the mGlu5 KO phenotype

In validating the mGlu5 KO mouse model, KO mice exhibited the expected phenotypes, including increased locomotion, reduced PPI and acoustic startle, and heightened acute sensitivity to a psychotropic compound, consistent with prior work [9, 10, 12, 24, 50]. Although KO mice showed reduced distance traveled in the LDB, this does not contradict their baseline hyperactivity; rather, because the LDB probes anxiogenic approach–avoidance conflict [51, 52], it may reflect reduced exploration or increased avoidance, consistent with our finding of reduced light exploration and prior reports of anxiety-like behavior in mGlu5 KO mice [53, 54]. This interpretation aligns with clinical observations that anxiety is highly prevalent and comorbid in patients with schizophrenia [2]. KO mice did not differ from WT controls in Porsolt immobility, partially aligning with Cai et al. [55], who found no baseline difference before repeated stress exposure.

### Acute psilocybin-induced behavioral responses

Psilocybin (1 mg/kg, i.p.) increased locomotor activity in mGlu5 KO but not WT mice, indicating that constitutive deletion of mGlu5 amplifies psilocybin’s stimulatory effects. This may reflect altered glutamatergic–serotonergic and, indirectly, dopamine signaling within cortico-striatal circuits; however, neurochemical levels were not directly assessed. Acute psilocybin also enhanced HTRs in male KO mice. However, this sex-specific contrast should be interpreted cautiously because female WT mice showed higher HTRs than male WT mice, reducing the detectable KO–WT difference in females. HTRs did not differ between male and female KO mice, and follow-up animals receiving a second psilocybin injection showed a similar enhancement in both sexes, suggesting broadly enhanced 5-HT_2A_-coupled behavioral responsivity in the absence of mGlu5. Because plasma and brain psilocin were not measured, exposure differences cannot be excluded; pharmacokinetic and multi-dose studies are needed. The second psilocybin challenge, given two weeks later, did not significantly differ from the first, suggesting no lasting behavioral sensitization under these conditions, consistent with chronic dosing findings [40]. Overall, enhanced locomotion and HTRs support the view that mGlu5 constrains 5-HT_2A_-mediated psychedelic responses [24]; however, neurodevelopmental adaptations cannot be excluded. An important future direction will be to test whether acute pharmacological blockade of mGlu5 in WT mice recapitulates these effects, which would help distinguish acute mGlu5-mediated regulation of psilocybin responses from compensatory adaptations arising from constitutive mGlu5 deletion.

### Neural correlates of psilocybin’s acute effects

Psilocybin increased claustral c-Fos in WT but not KO mice, suggesting that intact mGlu5 signaling contributes to psilocybin-evoked recruitment of this integrative node. This does not imply that greater claustral activation directly produces larger HTRs; indeed, KO mice showed enhanced acute behavioral responses, including increased locomotion and male-specific amplification of HTR, despite blunted claustral neuronal activation. Rather, the claustrum is thought to coordinate distributed cortical activity underlying perceptual and integrative aspects of the psychedelic state [36, 56, 57]. Consistent with its engagement in psychedelic signaling, local infusion of psilocin into the claustrum produces marked neurochemical changes and hallucinogen-related behaviors in rodents, supporting its role as a key node in psychedelic action [58]. Within this framework, reduced claustral recruitment in KO mice may reflect altered engagement of claustral/claustrocortical circuitry during psilocybin exposure, which could plausibly contribute to the altered acute behavioral responses observed in mGlu5 KO mice. However, this interpretation remains speculative and should be causally tested in future studies.

Dorsal striatal analyses further suggested genotype-dependent circuit alterations. mGlu5 KO mice showed reduced c-Fos density in the DMS but not DLS, consistent with the DMS’s stronger role in associative and goal-directed control and the DLS’s role in sensorimotor or habit circuitry [38, 59]. Elevated baseline locomotion in KO mice, together with reduced DMS c-Fos, may therefore reflect altered recruitment of associative or novelty-responsive cortico-striatal networks, rather than a simple increase in locomotor drive. This interpretation is consistent with reports of impaired locomotor habituation in mGlu5 KO mice [24, 60], where sustained activity may reflect reduced updating of environmental familiarity. At the level of the total dorsal striatum, there was a trend-level effect of psilocybin, suggesting modest engagement of this region. However, this did not resolve at the subregional level, where psilocybin did not significantly alter c-Fos density in either DMS or DLS. Accordingly, any link between psilocybin-induced hyperlocomotion and dorsal striatal activation remains tentative.

We did not detect an effect of psilocybin in the prelimbic cortex despite prior evidence that 5-HT_2A_ activation in this brain region can elicit the HTR in rats [37]. This may reflect species differences, mixed engagement of excitatory and inhibitory populations, and the temporal limits of a 90-min c-Fos readout. To help resolve some of these limitations, future work could use earlier markers such as pERK/pCREB and cell-type-specific c-Fos co-labeling.

### Post-acute and enduring effects of psilocybin on anxiety-like, depressive-like and psychotic-like behaviors

We found, in alignment with previous findings from our laboratory in different mouse models, that psilocybin administration at 1 mg/kg failed to attenuate the high-anxiety phenotype in mGlu5 KO mice using the LDB test [39, 41]. Unexpectedly, psilocybin increased immobility time in the Porsolt test in mice up to 7 days post-treatment. While this is typically interpreted as a depressive-like response, an alternative explanation is that immobility behavior may have reflected adaptive learning or energy conservation, particularly given our use of a baseline session prior to treatment [61, 62]. Interestingly, in males, greater psilocybin-induced HTR was not associated with immobility time. In females, however, greater psilocybin-induced HTR at the day 7 time point was associated with reduced immobility time. This pattern suggests  the group-level increase may reflect learning-related adaptations or other downstream processes not indexed by the HTR. Additional assessment using complementary assays of depressive-like and anxiety-like behaviors (e.g., saccharin-preference test, novelty-suppressed feeding test, elevated-plus maze) will help determine whether psilocybin influences anxiety-like or depressive-like behavior beyond the LDB and Porsolt test.

To the best of our knowledge, the enduring effects of a single psilocybin dose on subsequent PPI performance have not yet been characterized. Here, psilocybin produced a sustained sex-specific improvement in sensorimotor gating that was evident in female mGlu5 KO mice and was not observed in males. While we do not interpret this as evidence that psilocybin is broadly antipsychotic or that the effect generalizes across schizophrenia-related phenotypes, it does suggest that psilocybin can improve one schizophrenia-relevant endophenotype under conditions of mGlu5 dysfunction in females. The sex-specificity of this effect also highlights the need to consider sex as a biological variable in preclinical psychedelic research [63] and aligns with a growing body of work showing sex-specific behavioral effects of psychedelics [41, 64–69].

Additionally, we found that in female but not male animals, those that showed greater psilocybin-induced HTRs exhibited poorer PPI performance at 9 days after psilocybin administration. Although the female PPI effect differs in direction from the enhanced acute sensitivity observed in mGlu5 KO mice, we do not view these findings as contradictory; rather, they support the possibility that acute psilocybin sensitivity and longer-term effects on sensorimotor gating are mechanistically dissociable. Thus, an informative next experiment would be to test whether pretreatment with a selective 5-HT_2A_ receptor antagonist (to abolish the HTR) prevents, spares, or modifies psilocybin’s enduring improvement in PPI, and whether PPI effects are preserved across doses that differentially engage the HTR. Future studies should determine whether the sex differences observed here reflect sex-dependent serotonergic receptor biology. Prior work has shown region-specific sex differences in 5-HT_1A_ and 5-HT_2A_ receptor expression [70], estrogen-dependent increases in 5-HT_2A_ receptor density [71], and ovarian hormone-dependent regulation of 5-HT_2A/2C_ receptor expression [72]. For instance, previous research has found that female C57BL/6J mice exhibited greater DOI-induced head-twitch responding than males and showed slightly lower frontal cortical 5-HT_2A_ receptor density [73]. In the present study, females were not all born at exactly the same time and were counterbalanced across treatment groups; therefore, any uncontrolled estrous-stage variation would be expected to distribute across groups rather than systematically confound a single treatment condition. Nevertheless, recent evidence has shown that an uncontrolled estrous cycle stage does not necessarily increase overall behavioral variability in female mice [74]. Glutamatergic mechanisms may also contribute, as sex-dependent mGlu1/5 signaling has been reported in mice [75].

### Translational implications

Together, these findings indicate that mGlu5 loss alters both the acute behavioral sensitivity to psilocybin and its longer-lasting effects on schizophrenia-relevant sensorimotor gating, with important sex-dependent features. By combining acute measures of psychedelic-like responding with post-acute assessment of anxiety-like, depressive-like, and psychotic-like behaviors, the present study identifies mGlu5 as a potential modifier of serotonergic psychedelic sensitivity. The enhanced locomotor and HTR responses in mGlu5 KO mice suggest that loss of mGlu5 can amplify 5-HT_2A_-coupled behavioral output, whereas the sustained rescue of PPI in female KO mice indicates that psilocybin can also engage longer-lasting mechanisms relevant to schizophrenia-related endophenotypes. This dissociation between acute psychedelic-like output and later sensorimotor-gating effects is a key strength of the study.

 The findings of the present study  may have relevance to schizophrenia, where glutamatergic dysregulation is a prominent pathophysiological feature [4] and dopamine–glutamate interaction models remain central [76]. The genotype-dependent locomotor response may plausibly involve altered cortico-striatal regulation of dopamine, although dopaminergic function was not directly assessed. At the same time, the female-specific improvement in PPI suggests that psilocybin can induce longer-lasting changes in sensorimotor gating under conditions of mGlu5 dysfunction. Given psilocybin’s serotonergic pharmacology and the glutamatergic nature of the model, the observed PPI improvement is consistent with prior evidence implicating serotonin–glutamate interactions in schizophrenia-relevant endophenotypes [77–79], while dopaminergic contributions remain possible [80]. This broader mechanistic framing may be particularly relevant for treatment-resistant populations, in which D_2_-targeting strategies are often insufficient and glutamatergic abnormalities may be more prominent [81].

The enhanced sensitivity to psilocybin observed in mGlu5 KO mice may also have translational relevance for clinical populations with schizophrenia or psychosis vulnerability. Heightened locomotor activity and HTRs in mGlu5 KO mice could reflect increased susceptibility to schizophrenia-like or destabilizing effects, supporting caution in the use of serotonergic psychedelics in individuals at risk for psychosis. In line with this, historical clinical observations suggest that individuals with schizophrenia may show more severe or psychotic-like acute responses to psilocybin than healthy volunteers [82]. However, because the HTR is a preclinical proxy of 5-HT_2A_ receptor activation rather than a direct measure of subjective psychedelic experience, amplified 5-HT_2A_-linked behavioral responses should not necessarily be interpreted as negative or pathological. In humans, acute psilocybin effects include perceptual and mystical-type experiences [83], and the intensity of these effects has been shown to correlate with 5-HT_2A_ receptor occupancy and plasma psilocin levels [84]. Such subjective effects have also been proposed to contribute to enduring therapeutic outcomes [85].

From a precision medicine perspective, these findings raise the possibility that individuals carrying rare or functional variants in GRM5, the gene encoding mGlu5, may exhibit altered sensitivity to serotonergic psychedelics. The sex-specific findings observed here further support a personalized approach, suggesting that psychedelic responses in the context of mGlu5 dysfunction may need to be considered separately in males and females.

## Conclusion

Overall, our study shows for the first time that a loss of mGlu5 signaling results in an amplified acute sensitivity to psilocybin—particularly in males—manifested as increased locomotor activity and HTRs, while psilocybin-evoked claustral c-Fos was observed only in WT, not KO mice. These findings suggest that mGlu5 constrains 5-HT_2A_-coupled signaling and supports coordinated claustral recruitment. In contrast, psilocybin produced a longer-lasting, sex-specific rescue of PPI in female KO mice, and correlational analyses indicate that acute psychedelic-like output and later sensorimotor-gating effects may be dissociable. The mGlu5 KO mouse model therefore provides a useful platform for dissecting serotonergic–glutamatergic mechanisms relevant to schizophrenia-related phenotypes and for testing strategies that preserve schizophrenia-relevant behavioral benefits while minimizing hallucinogenic-like liability.

## Supplementary information

Supplementary Material including Figures and Tables

Supplementary Table 3

## Data Availability

The raw data supporting the findings of this study, together with the analysis code used to generate the reported results, are available from the corresponding author upon reasonable request.
