# Supplementary material for: Psilocybin produces amplified acute responses and sex-specific long-term increases in sensorimotor gating in the mGlu5 knockout mouse model of schizophrenia
Source: Neuropsychopharmacology. 2026 Jul 18;51(11):2111–23. doi: 10.1038/s41386-026-02501-3 (PMC13598084; doi:10.1038/s41386-026-02501-3)
Supplement: Supplementary file 1 [file 41386_2026_2501_MOESM1_ESM.pdf]

Supplementary file

Psilocybin produces amplified acute responses and sex-specific long-term increases in sensorimotor gating in the mGlu5 knockout mouse model of schizophrenia

James J Gattuso[a,b], Ahmed Kamal[a,c], Jennyfer M. Payet[d], Kato Havaux[a], Jenna Hendey[a], Bilgenur Bezcioglu[a,c], Zoe J. Phelan[d], Maddy Orchard[a,c], Divyesh Bhanot[a,c], Matthew W. Hale[d], Anthony J. Hannan[a,b,c,*], Thibault Renoir[a,b,e*]

[a]Florey Institute of Neuroscience and Mental Health, Parkville, Australia.

[b]Florey Department of Neuroscience and Mental Health, University of Melbourne, Parkville, Australia.

[c]Department of Anatomy and Physiology, University of Melbourne, Parkville, Australia.

[d]Department of Psychology, Counselling and Therapy, School of Psychology and Public Health, La Trobe University, Melbourne, Victoria, Australia

[e]Academic Quality Department, Melbourne Polytechnic, Australia.

[*]Joint last authors

Correspondence to: Dr Thibault Renoir or Professor Anthony J. Hannan, Florey Institute of Neuroscience and Mental Health, University of Melbourne, VIC 3010, Melbourne, Australia. thibault.renoir@florey.edu.au or anthony.hannan@florey.edu.au

Supplementary Methods

Pre-pulse inhibition (PPI)

Sensorimotor gating was assessed using PPI with an automated SR-Lab startle response system (San Diego Instruments, San Diego, CA), following established protocols [1,2]. Testing was conducted in sound-attenuated chambers, with acoustic stimuli delivered over a continuous 70-dB background noise via ceiling-mounted speakers. Startle responses were elicited by 115-dB pulses lasting 40 ms and recorded in millivolts (mV) using a piezo-electric accelerometer located beneath the testing platform.

Each PPI session comprised 104 trials. The initial and final eight trials were 115-dB startle pulse-only trials (P115). The remaining trials included 16 additional P115 trials, eight no-stimulus trials to assess baseline activity, and 64 prepulse–pulse trials. In prepulse–pulse trials, a 20-ms non-startling prepulse was presented either 30 or 100 ms before the startle pulse, at intensities of 2, 4, 8, or 16 dB above the 70-dB background noise. These conditions are referred to as PP2P115, PP4P115, PP8P115, and PP16P115, respectively. Trial order was pseudorandomised, and inter-trial intervals varied randomly between 10 and 37 s.

The percentage of PPI (%PPI) was calculated by subtracting the median startle amplitude in prepulse–pulse trials from the median amplitude in pulse-only trials, dividing by the pulse-only value, and multiplying by 100. Sensorimotor gating was assessed at both 30- and 100-ms prepulse–pulse intervals, as PPI varies as a function of interstimulus interval (ISI), and different ISIs can show differential sensitivity to underlying neurotransmitter mechanisms and pharmacological manipulations [3].

Because baseline PPI differs across inbred strains, with C57BL/6J mice often exhibiting relatively low PPI [4], all genotype and drug effects were evaluated within the same C57BL/6J

background relative to same-cohort vehicle controls tested under identical conditions, consistent with previous studies using mGlu5-deficient mice on this background [2,5].

c-Fos Immunohistochemistry and quantification

90 minutes following psilocybin or saline administration, the mice were anaesthetized with pentobarbital (100 mg/kg; Virbac) in 0.9% saline i.p. After the mice were anaesthetized, they were transcardially perfused with phosphate buffered saline (PBS) for 4 minutes followed by 4% paraformaldehyde (PFA) solution for 4 minutes. The brains were then stored in 4% PFA overnight. Following this, the brain was placed in 30% sucrose in PBS for approximately 7 days (all brains had sunk). Brains were fast frozen using isopentane on dry ice and then stored in a −80 °C freezer. Coronal sections (30 µm) were then embedded in OCT cut from +2.0 mm to –5.0 mm using a cryostat (–22 °C; Leica) into 24-well tissue culture plates and stored in a −20 °C freezer in cryoprotectant solution.

Peroxidase immunohistochemistry for c-Fos expression analysis was then conducted as previously described [6]. Free-floating immunohistochemistry was performed in 0.05 M phosphate buffered saline (PBS) with gentle orbital agitation. Endogenous peroxidase was quenched in 1% H_2O_2 prepared in 50% methanol/50% PBS (30 min), followed by PBS rinses and permeabilization in PBST (0.3% Triton X-100 in PBS, 30 min). Sections were incubated overnight at room temperature with mouse anti-c-Fos primary antibody (1:1000; Santa Cruz, sc-271243) in 0.3% PBST. The next day, sections were rinsed, incubated with biotinylated goat anti-mouse secondary antibody (1:500; Vector, BA-9200, 90 min), and developed using the VECTASTAIN® Elite ABC-Peroxidase kit (Vector, PK-6100; ABC prepared ≥30 min before use) followed by Vector SG chromogen (Vector, SK-4700) for 10–20 min with microscopic monitoring to standardize reaction endpoints. After PBS rinses, sections were stored at 4 °C short-term or returned to cryoprotectant at –20 °C.

For mounting, sections were arranged onto StarFrost uncoated slides (ProSciTech, G311SF) using a fine brush, briefly coated with 0.15% gelatin (Type A, ~175 g bloom; Sigma, G2625), and air-dried overnight. Slides were dehydrated through graded ethanol (70%, 95%, 100%), cleared in xylene, and coverslipped with DPX. Whole-slide brightfield images were acquired on an Axioscan 7 (Zeiss) at 10× with standardized settings (0.3457 µm/pixel; 8-bit RGB, .czi). Image analysis was performed in QuPath (v0.6.0, 64-bit).

Regions of interest were defined a priori based on their established involvement in psychedelic-related mechanisms, relevance to schizophrenia pathophysiology, and/or their functional linkage to the behavioral outcomes in which significant genotype × treatment effects were observed. We selected the medial prefrontal cortex (mPFC; including prelimbic and infralimbic subregions) due to its central role in serotonergic psychedelic action. Activation of 5-HT_{2A} receptors within the mPFC is sufficient to induce the HTR [7], a canonical proxy of psychedelic-like effects in rodents. Furthermore, psilocybin has been shown to promote dendritic spine growth in the mPFC [8], consistent with its proposed plasticity-enhancing effects, and structural and functional abnormalities in this region are well documented in schizophrenia [9]. The dorsal striatum was included due to its critical role in regulating locomotor activity [10] and mediating responses to dopaminergic/psychostimulant drugs [11,12], making it directly relevant to the hyperlocomotor phenotype and psilocybin induced hyperlocomotion observed in mGlu5 KO mice. Finally, the claustrum was selected as a key region of interest given its high expression of 5-HT_{2A} receptors in both humans [13] and rodents [14] and emerging evidence implicating it as a central node in the subjective and integrative effects of psychedelics in humans [15]. Finally, the claustrum has also been implicated in schizophrenia psychopathology [16].

For each region, standardized bilateral regions of interest (ROIs) were defined. The claustrum was identified using established anatomical landmarks, including the white matter tract of the corpus callosum (Supplementary Figure 4), and ROI boundaries were defined conservatively to minimise encroachment into adjacent structures. Claustrum sections were sampled across a rostrocaudal range from bregma +1.94 to −0.22 mm. Given the substantial anteroposterior extent of the claustrum, 1–3 sections were analysed per animal and values were averaged to provide a representative measure across the region. Across animals, an average of 2.21 sections per mouse contributed to the claustrum analysis. In cases where only a single section was included, additional sections were excluded due to technical issues (e.g., tissue tearing, damage, or high background signal) that precluded reliable c-Fos quantification. These exclusions were distributed across experimental groups.

c-Fos-positive nuclei were quantified using the Positive Cell Detection module with fixed parameters across all slides: requested pixel size 0.5 µm, background radius 8.0 µm, median filter radius 0.8 µm, sigma 1.5 µm, DAB OD threshold 0.30, watershed post-processing enabled, cell expansion 2.0 µm, circularity ≥0.40, and size thresholds corresponding to ~6–239 μm^2 (50–2000 px^2 at 0.3457 µm/pixel). Automated detections were verified by systematic visual inspection. "Ignore/Exclusion" annotations were applied to remove artefacts and non-neuronal regions. If background artifacts were substantial, manual cell counting by a blinded researcher was substituted. For each animal, counts (cells/mm^2) were averaged across sections and across both hemispheres.

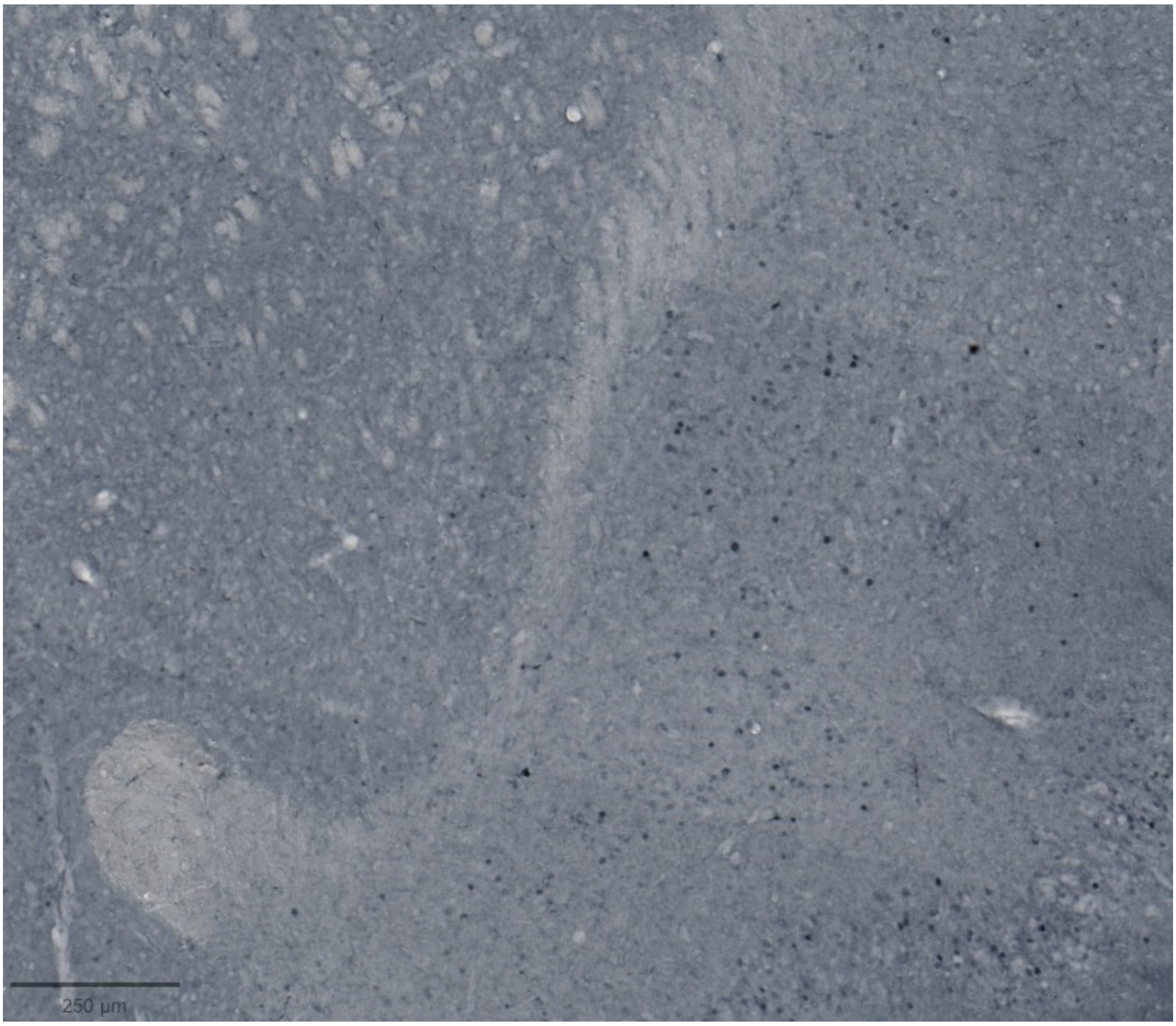

Supplementary Figure 1.
Representative c-Fos immunoreactivity in a coronal section at approximately bregma +1.34 mm, encompassing the claustrum and surrounding cortex (left hemisphere). Dark punctate nuclei represent c-Fos–positive cells. Scale bar = 250 µm.

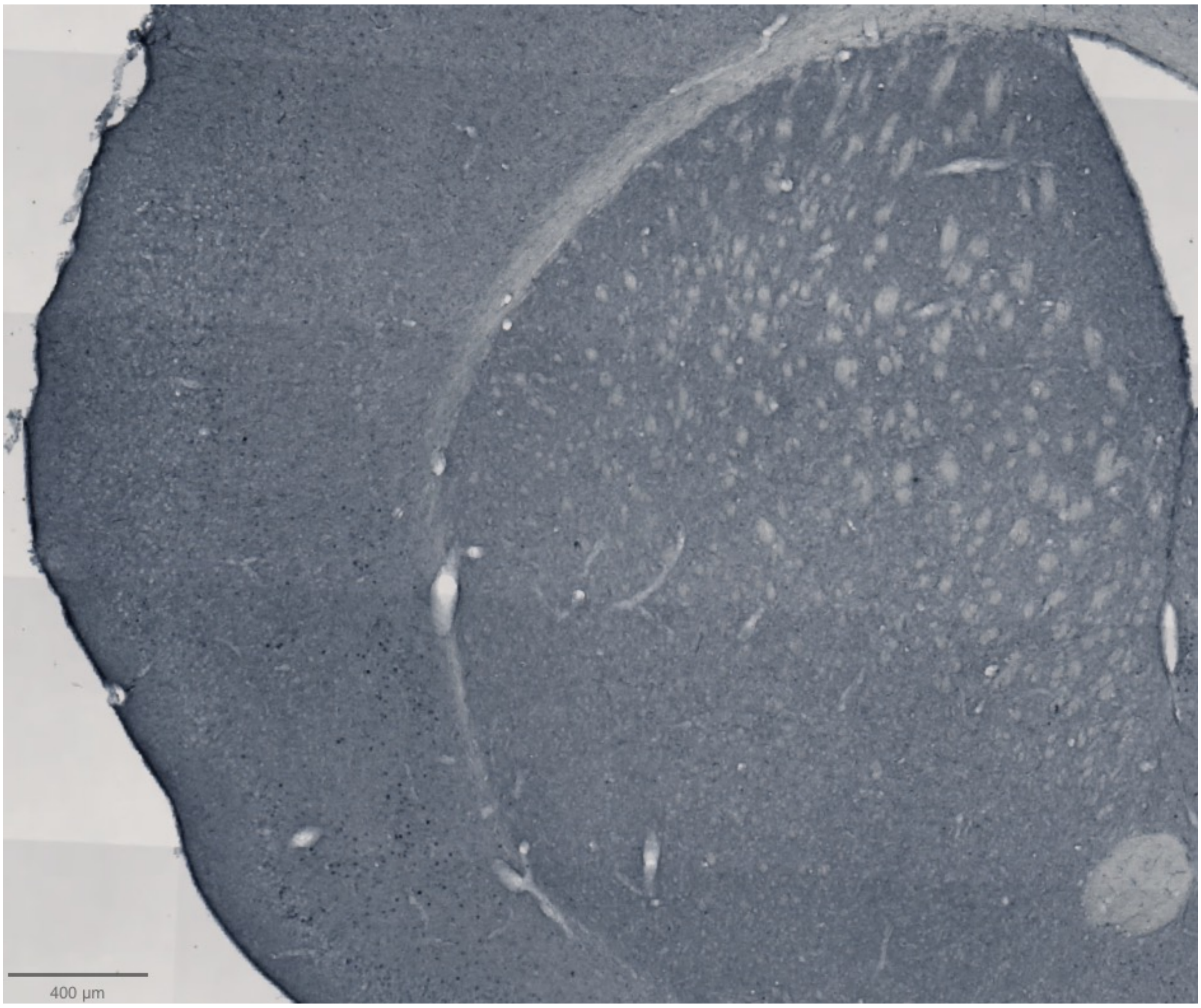

Supplementary Figure 2.
Representative c-Fos immunoreactivity in a coronal section at approximately bregma +0.38mm, encompassing the dorsal striatum and surrounding cortex (left hemisphere). Dark punctate nuclei represent c-Fos–positive cells. Scale bar = 400 µm.

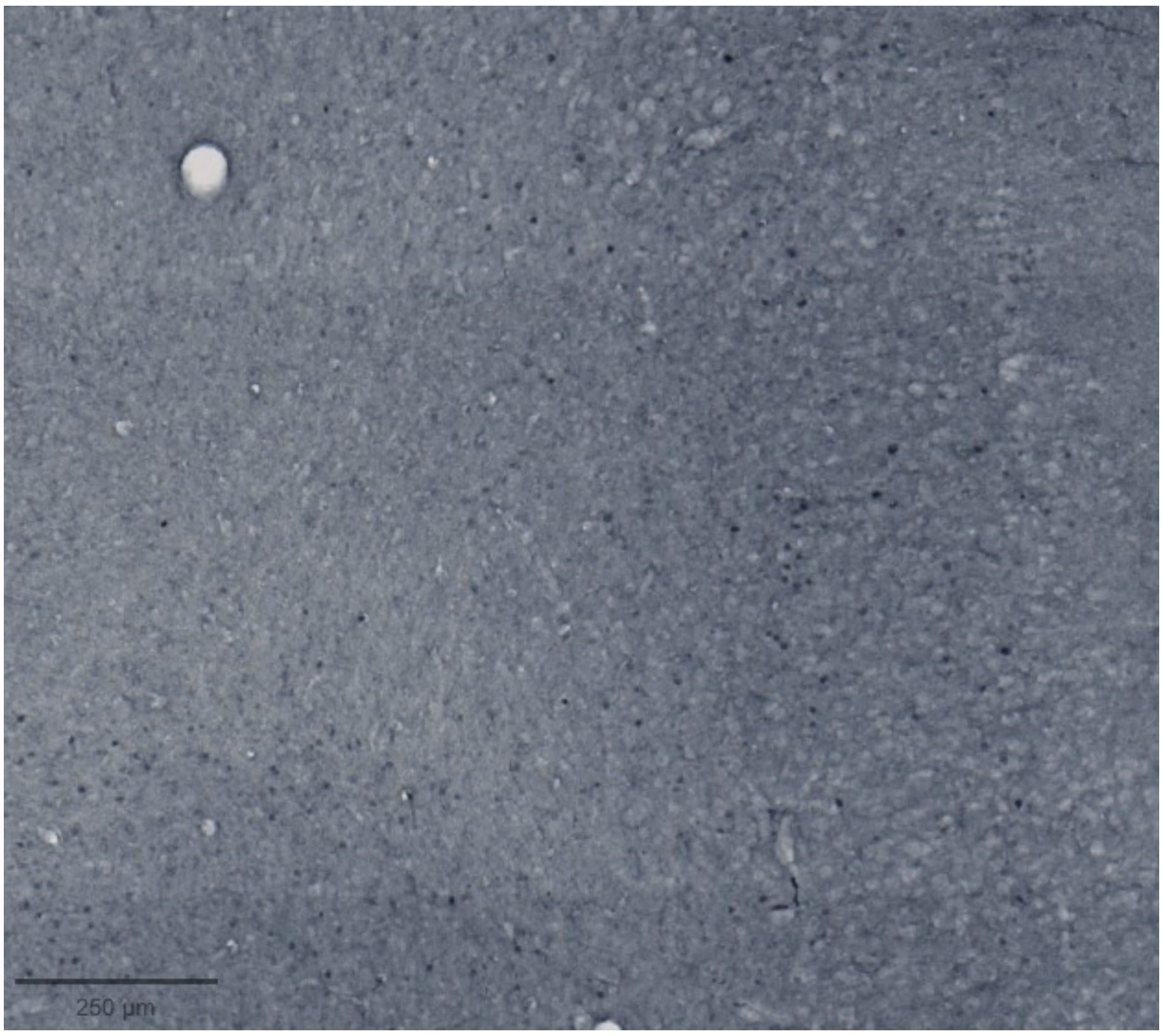

Supplementary Figure 3.
Representative c-Fos immunoreactivity in a coronal section at approximately bregma +1.98, encompassing the mPFC and surrounding cortex (left hemispheres). Dark punctate nuclei represent c-Fos–positive cells. Scale bar = 250 µm.

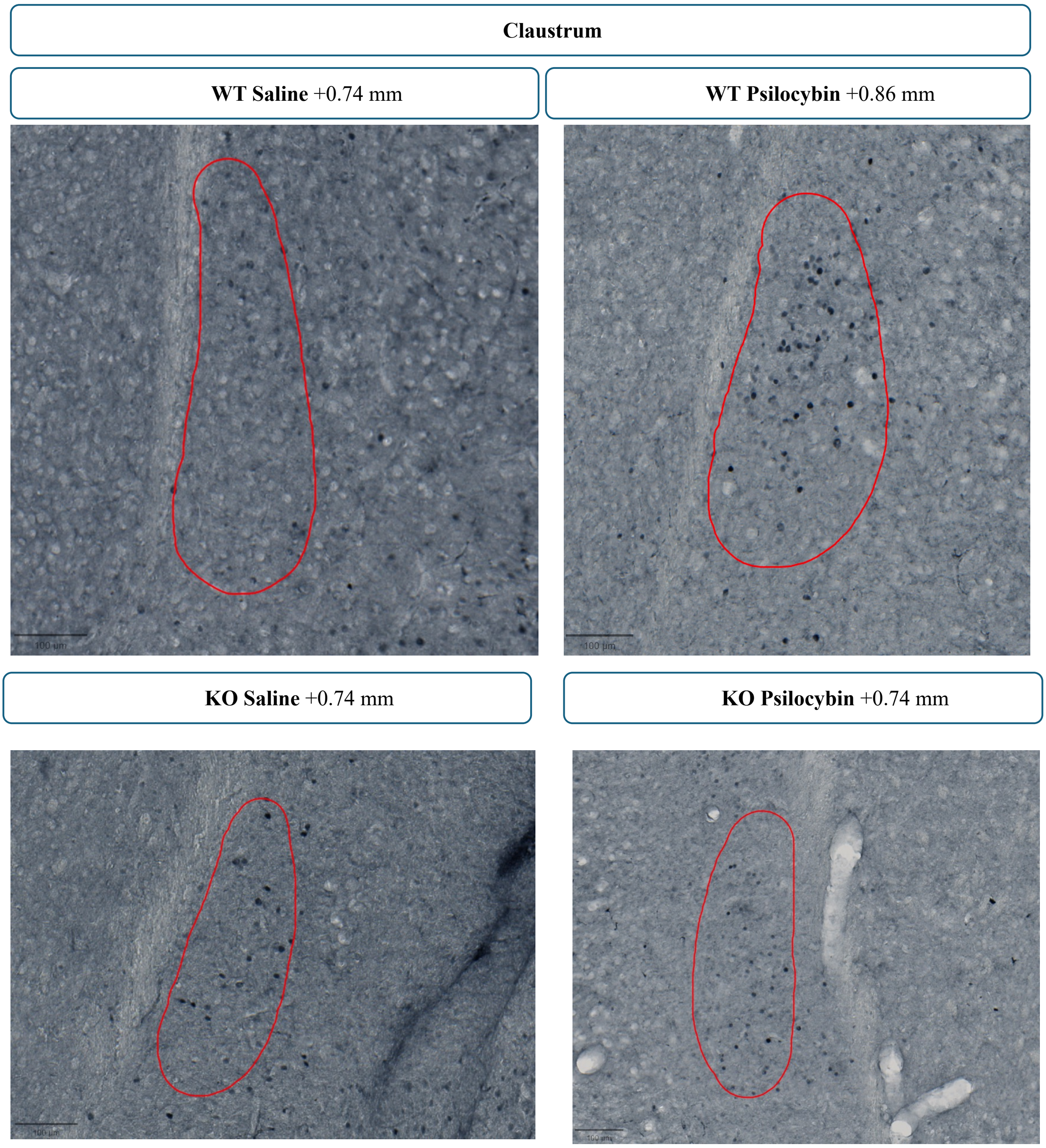

Supplementary Figure 4. c-Fos immunoreactivity in the claustrum at high magnification. Representative images of c-Fos–positive cells within the claustrum from WT and mGlu5 KO mice following saline or psilocybin administration. These images illustrate the cellular-level differences underlying the group effects reported in the main analysis. The claustrum is outlined in red based on anatomical landmarks as described in the Methods. Images are shown at comparable rostrocaudal levels relative to bregma (WT Saline: +0.74 mm; WT Psilocybin: +0.86 mm; KO Saline: +0.74 mm; KO Psilocybin: +0.74 mm). Consistent with the quantitative analyses, psilocybin markedly increased c-Fos expression in the claustrum of WT mice compared to saline controls, whereas no difference was observed between saline- and psilocybin-treated mGlu5 KO mice. Scale bar = 100 µm.

Statistical analysis

All statistical analyses were conducted using GraphPad Prism version 10.4.2 or R version 4.4.2. Graphs were generated using GraphPad Prism or RStudio version 2026.01.1+403. A significance threshold of $p < 0.05$ was applied to all comparisons, and a statistical trend was defined when $0.05 \leq p \leq 0.06$. For acute locomotor activity and HTR, three-way analyses of variance (ANOVAs) were conducted with treatment, sex, and genotype included as factors. For primary post-acute behavioral outcomes, statistical analyses were conducted using linear mixed-effects models in R. Outcome measures were modeled as the dependent variable, with genotype (WT, mGlu5 KO), treatment (saline, psilocybin), sex (male, female), time (days post-injection), and their relevant interactions included as fixed effects. Mouse ID was included as a random intercept to account for within-subject correlations across repeated measurements. Models were fitted using maximum likelihood estimation. Type III analyses of variance were used to evaluate main effects and interactions, with denominator degrees of freedom estimated using Satterthwaite's approximation. Where significant interaction effects were identified, post hoc comparisons were conducted using estimated marginal means to interrogate simple effects consistent with the structure of the interaction. These analyses were performed using the *lme4* [17] and *lmerTest* [18] packages for model fitting and inference, and the *emmeans* package [19] for estimation of marginal means and post hoc comparisons.

For secondary post-acute behavioral measures, analyses were conducted separately within males and females. In these sex-stratified analyses, repeated-measures three-way ANOVAs were used in GraphPad Prism when complete repeated-measures data were available, with genotype, treatment, and time included as factors.

For the head-twitch response (HTR), although we detected a significant genotype × treatment × sex interactions we conducted a difference-in-differences (ΔΔ) analysis in R using a linear model to determine whether male or female mGlu5 KO mice had a greater increase in the HTR

compared to WT mice receiving psilocybin. $\Delta\Delta_{HTR}$ analyses were computed separately by sex: $[(KO - WT)_{psil,male} - (KO - WT)_{sal,male}]$ and $[(KO - WT)_{psil,female} - (KO - WT)_{sal,female}]$.

Because c-Fos analyses were based on smaller sample sizes ($n \leq 10$ per group), assumptions of normality and homogeneity of variance were formally evaluated prior to conducting ANOVAs, given that parametric tests are less robust to assumption violations with lower sample size. Normality of residuals was assessed using the Shapiro–Wilk test and Q–Q plots. Homogeneity of variance was assessed using (i) a Spearman rank test of heteroscedasticity (absolute residuals vs fitted values), (ii) inspection of residuals-versus-fitted and heteroscedasticity plots. When residuals deviated from normality, data were transformed (e.g., square-root) and models re-fitted. If residuals remained non-normal after transformation, we used a permutation ANOVA implemented in R with the package permuco [20] (function aovperm; sum-to-zero contrasts specified using options(contrasts = c("contr.sum","contr.poly")), 10,000 permutations, Freedman–Lane method, Type-III sums of squares, set.seed(12345)). When residuals were normally distributed, but variances were heterogeneous, we did not transform; instead we applied a Welch-type heteroscedastic ANOVA for factorial designs (R package welchADF [21], function welchADF.test). Planned or simple-effects contrasts under heteroscedasticity used Welch two-sample t-tests (t.test(var.equal = FALSE)).

To account for individual differences in baseline acoustic startle reactivity, a sensitivity analysis was conducted in which pulse-alone startle amplitude (StartlePA) was included as a covariate in the mixed-effects model. Prior to inclusion, startle values were mean-centered within each interstimulus interval (ISI), such that the covariate (StartlePA_c) reflected deviations from the sample mean startle response. This centering ensured that model intercepts and estimated marginal means were interpretable at the average level of startle reactivity. The

startle-adjusted model was specified identically to the primary model, with %PPI as the dependent variable and sex, genotype, treatment, and time included as fixed effects, alongside StartlePA_c as an additive covariate. Mouse ID was included as a random intercept to account for repeated measurements. Models were fitted separately within each ISI (30 ms and 100 ms), consistent with the primary analyses. Estimated marginal means and pairwise comparisons from the startle-adjusted model were evaluated at StartlePA_c = 0, corresponding to the mean pulse-alone startle amplitude within each ISI. This approach allowed group differences in %PPI to be interpreted independently of variability in baseline startle magnitude, thereby providing a more specific assessment of sensorimotor gating. For visualization, estimated marginal means from the startle-adjusted model were plotted using *ggplot2* in R studio [22].

Associations between the HTR and performance in the Porsolt Swim Test (PST) and PPI tests (Figures 4 and 5) were assessed using Pearson's correlation coefficient (*r*). Only psilocybin-treated mice were included, as saline-treated animals exhibit negligible or no head twitches; including them would introduce a floor effect in the HTR variable and limit the interpretability of correlation estimates. Therefore, restricting analyses to psilocybin-treated animals ensures the relationship reflects individual differences in 5-HT_{2A} receptor activation and its behavioral consequences. However, because genotype differences in HTR were observed in male mice, a separate analysis for males was conducted to account for potential confounding effects of genotype. For these analyses, linear regression models were implemented in R to examine the relationship between HTR and behavioral outcomes. Specifically, models of the form *behavior ~ HTR × genotype* were fitted to test whether the association between HTR and behavioral outcome differed between WT and mGlu5 KO mice. The significance of the HTR × genotype interaction term was used to determine whether genotype moderated the relationship between HTR and behavior. Where the interaction term was not significant, a reduced model (*behavior*

~ *HTR* + *genotype*) was used to estimate the genotype-adjusted association between HTR and behavioral outcome. To further quantify this relationship independently of genotype, partial correlations were computed using Pearson's method, controlling for genotype as a covariate. Accordingly, PPI correlational analyses were conducted separately for each ISI at each time point, whereas PST correlational analyses were conducted separately for each time point.

Post hoc comparisons were adjusted for multiple comparisons, where applicable, using Šidák or Holm–Bonferroni correction, as appropriate.

	**Psilocybin 1 mg/kg**		**Saline**		
	WT	KO	WT	KO	TOTAL
**males**	11	10	12	11	44
**females**	14	13	13	12	52
					96

Supplementary Table 1. The number of mice used in behavioral experiments.

	**Psilocybin 1 mg/kg**		**Saline**		
	WT	KO	WT	KO	TOTAL
**males**	10	8	8	9	35
**females**	7	6	6	7	26
					61

Supplementary Table 2. The number of mice used for c-Fos analysis. **Note:** In some brain regions, not all animals could be included in the analysis due to tissue damage in specific slices, which resulted in the absence of suitable sections at the bregma levels of interest.

Supplementary Results

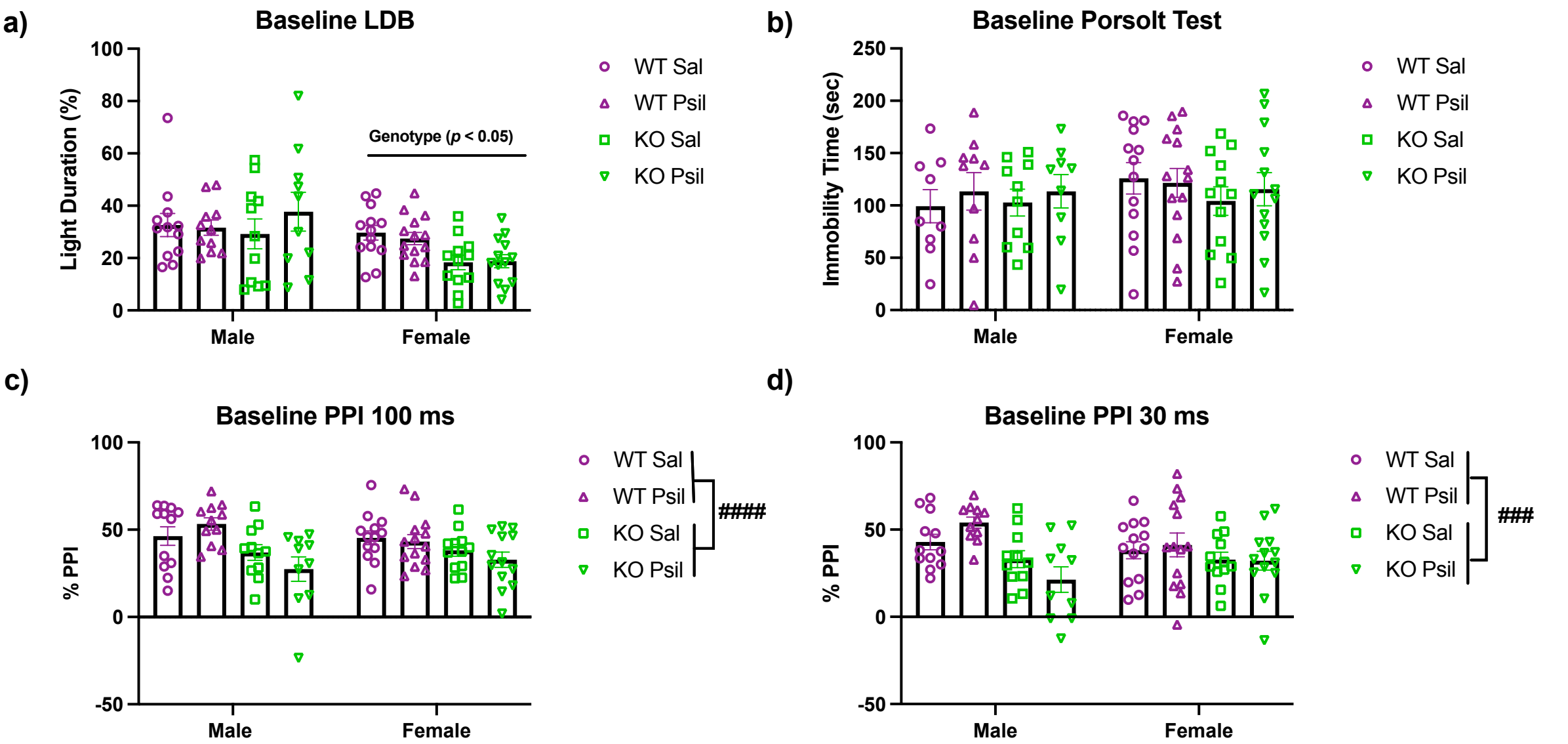

Supplementary Figure 5. Baseline behavioral results prior to treatment administration **a)** In the LDB, there was a significant sex × genotype interaction ($p = 0.045$), where post hoc analysis revealed that female KO mice spent less time in the light compartment compared to female WT mice ($p = 0.019$). There was no significant genotype effect in males ($p = 0.950$). **b)** For immobility time in the baseline Porsolt test, there were no significant effects. **c,d)** For PPI, there was a significant main effect of genotype, with KO mice showing reduced %PPI compared to WT mice at both 100 ms ($p < 0.0001$) and 30 ms ($p < 0.001$). Across all baseline behavioral measures, there were no significant differences between mice allocated to saline or psilocybin treatment groups (all $p \geq 0.436$). Sal and Psil indicate subsequent treatment allocation; all mice were treatment-naïve at baseline. For panels a, c, and d, n = 10–14 mice per group; for panel b, valid baseline Porsolt data were available for n = 9–14 mice per group. Bars represent mean ± SEM; individual data points represent mice. WT = wild-type, KO = knockout, Sal = saline, Psil = psilocybin 1 mg/kg. $^{###}p < 0.001$, $^{####}p < 0.0001$, WT vs KO. For panel a, bold text above grouped bars denotes the significant genotype effect within female mice.

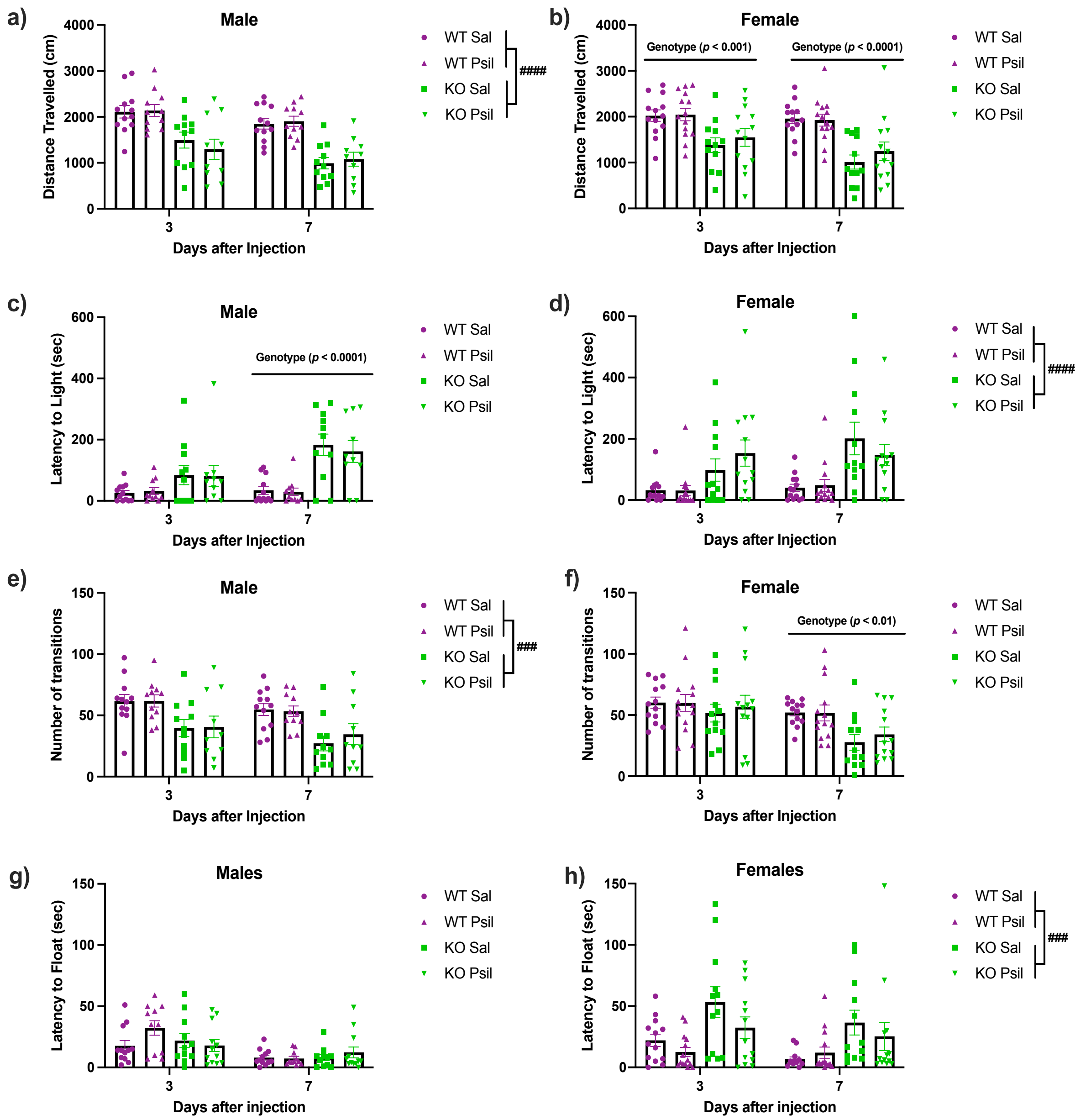

Supplementary Figure 6. Subsidiary measures for the light-dark box and Porsolt swim test.

a) For distance travelled in male mice in the LDB test, there was a significant main effect of time ($p < 0.0001$) and genotype ($p < 0.0001$), where mGlu5 KO mice travelled less than WT mice, but no significant effect of treatment ($p = 0.953$). There were no significant interactions. **b)** For distance travelled in the LDB for female mice there was a genotype × time interaction, where post hoc analysis revealed that mGlu5 KO mice travelled less than WT mice at both 3 ($p < 0.001$) and 7 days after injection ($p < 0.0001$). There was no significant effect of treatment ($p = 0.489$) and no significant interactions. **c)** For latency to the light compartment of the LDB in males, we found a significant time × genotype interaction ($p = 0.004$), where post hoc analysis revealed that male KO mice had greater latency to the light compartment compared to male WT mice 7 days after injection ($p < 0.0001$), but not 3 ($p = 0.055$). There was no effect of treatment ($p = 0.781$) or any other interactions. **d)** For female latency to light, there was a main effect of genotype ($p < 0.0001$), whereby female KO mice took longer to enter the light side of the LDB compared to female WT mice. There were no other significant effects. **e)** When

analyzing the number of transitions between the light and dark components of the LDB in male mice, we found that KO mice had fewer transitions compared to WT mice ($p < 0.001$) and there was a main effect of time ($p = 0.002$), however no effect of treatment ($p = 0.752$) or any significant interactions. **f)** For females, the number of transitions varied by genotype and time ($p = 0.018$), whereby mGlu5 KO mice had fewer transitions at day 7 ($p = 0.003$) but not day 3 ($p = 0.608$). There was no effect of treatment ($p = 0.645$) or any other interaction. **g)** We assessed the latency to float in the PST for male mice and found a time × genotype × treatment interaction reached significance ($p = 0.034$), however, there were no significant post hoc results. **h)** For female mice mGlu5 KO mice exhibited greater latency to float compared to WT mice ($p < 0.001$) and there was also a significant effect of time ($p = 0.046$). There was no effect of treatment ($p = 0.056$) or any interactions. n = 10 – 14 mice per group. Bars represent mean ± SEM; individual data points represent mice. WT = wild-type, KO = knockout, Sal = saline, Psil = psilocybin 1 mg/kg. $^{###}p < 0.001$, $^{####}p < 0.0001$, WT vs KO. For panels b, c, and f, bold text above grouped bars denotes significant genotype effects at the specified day 3 or day 7 comparison.

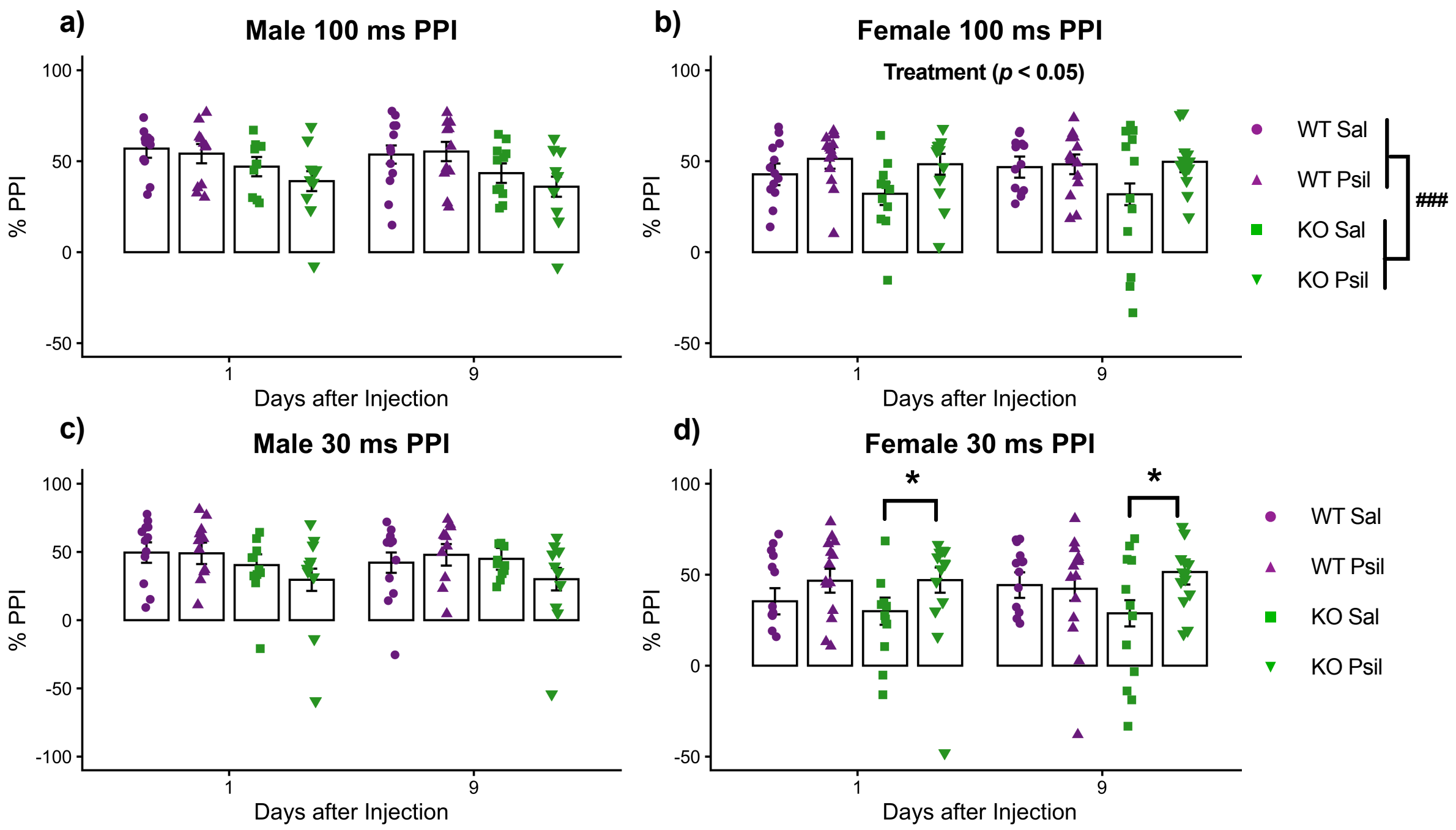

Supplementary Figure 7. Startle-adjusted PPI following psilocybin treatment (a,b) For 100 ms PPI both male and female mGlu5 KO mice exhibited significantly less PPI compared to WT mice ($p < 0.001$). Furthermore, a sex × treatment interaction was found ($p = 0.018$), and post hoc analysis found that female mice receiving psilocybin had greater PPI compared to those receiving saline ($p = 0.014$). **(c, d)** For 30 ms PPI a significant sex × treatment × genotype effect was observed ($p = 0.048$). Post hoc analysis found that female KO mice receiving psilocybin had significantly greater PPI compared to saline ($p = 0.018$), whereas there was no treatment effect for WT mice ($p = 0.828$). For males, psilocybin did not alter PPI in either WT or KO mice ($p = 0.793$ & $p = 0.137$ respectively). Bars represent estimated marginal means derived from covariate-adjusted linear mixed-effects models, with error bars indicating ± SEM; individual points represent raw values for each mouse. n = 10–14 mice per group. WT = wild-type, KO = knockout, Sal = saline, Psil = psilocybin 1 mg/kg. $^{*}p < 0.05$, KO Sal vs KO Psil. $^{###}p < 0.001$, WT vs KO. For panel b, the bold text above the groups represents a significant main effect of treatment.

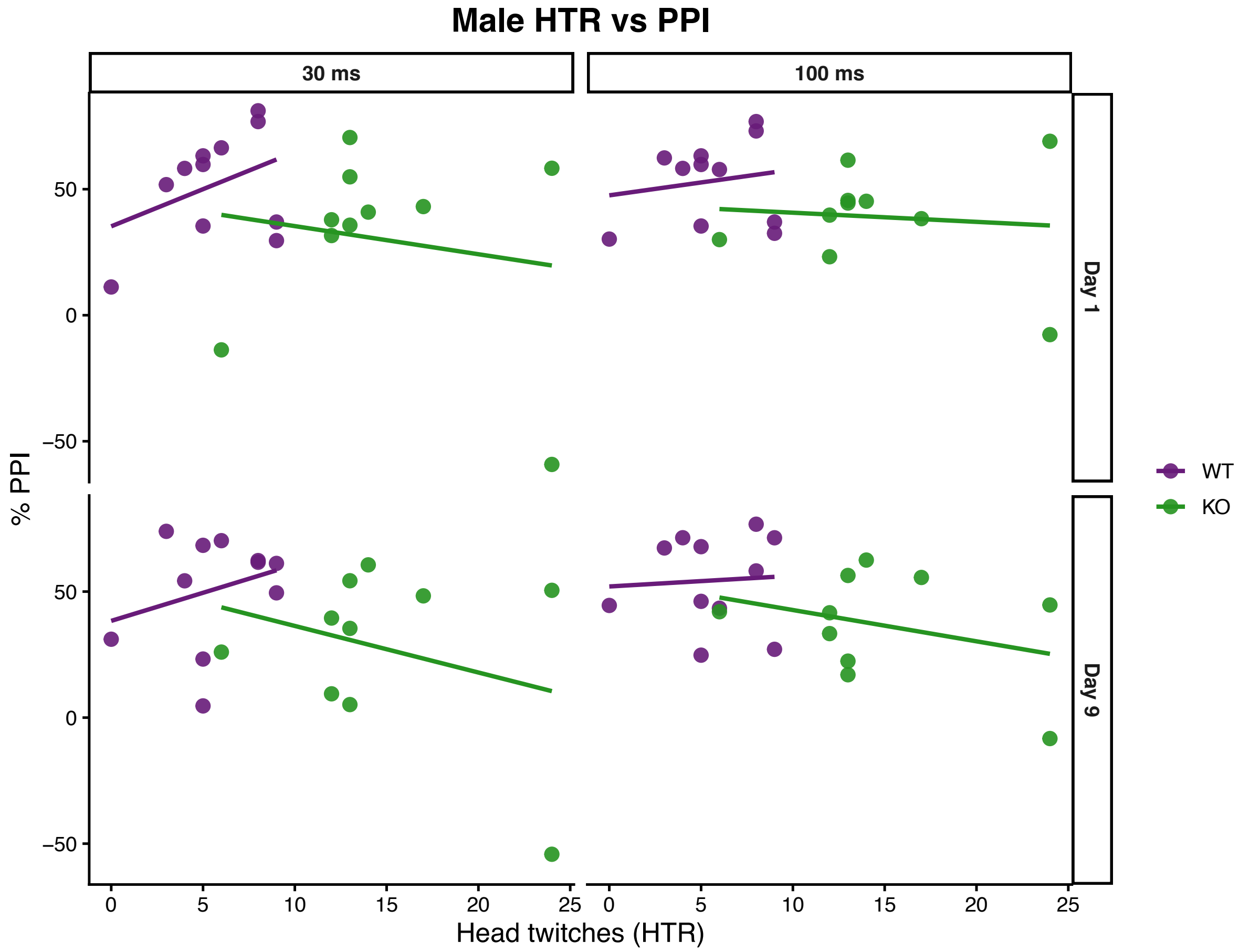

Supplementary Figure 8. Relationship between psilocybin-induced HTR and PPI in male mice. Scatterplots show HTR plotted against %PPI at 30 ms and 100 ms prepulse intervals on day 1 and day 9 after psilocybin administration in male WT and mGlu5 KO mice. Each point represents an individual mouse; lines indicate genotype-specific linear fits. *n* = 21 (11 WT, 10 KO). WT = wildtype, KO = knockout.

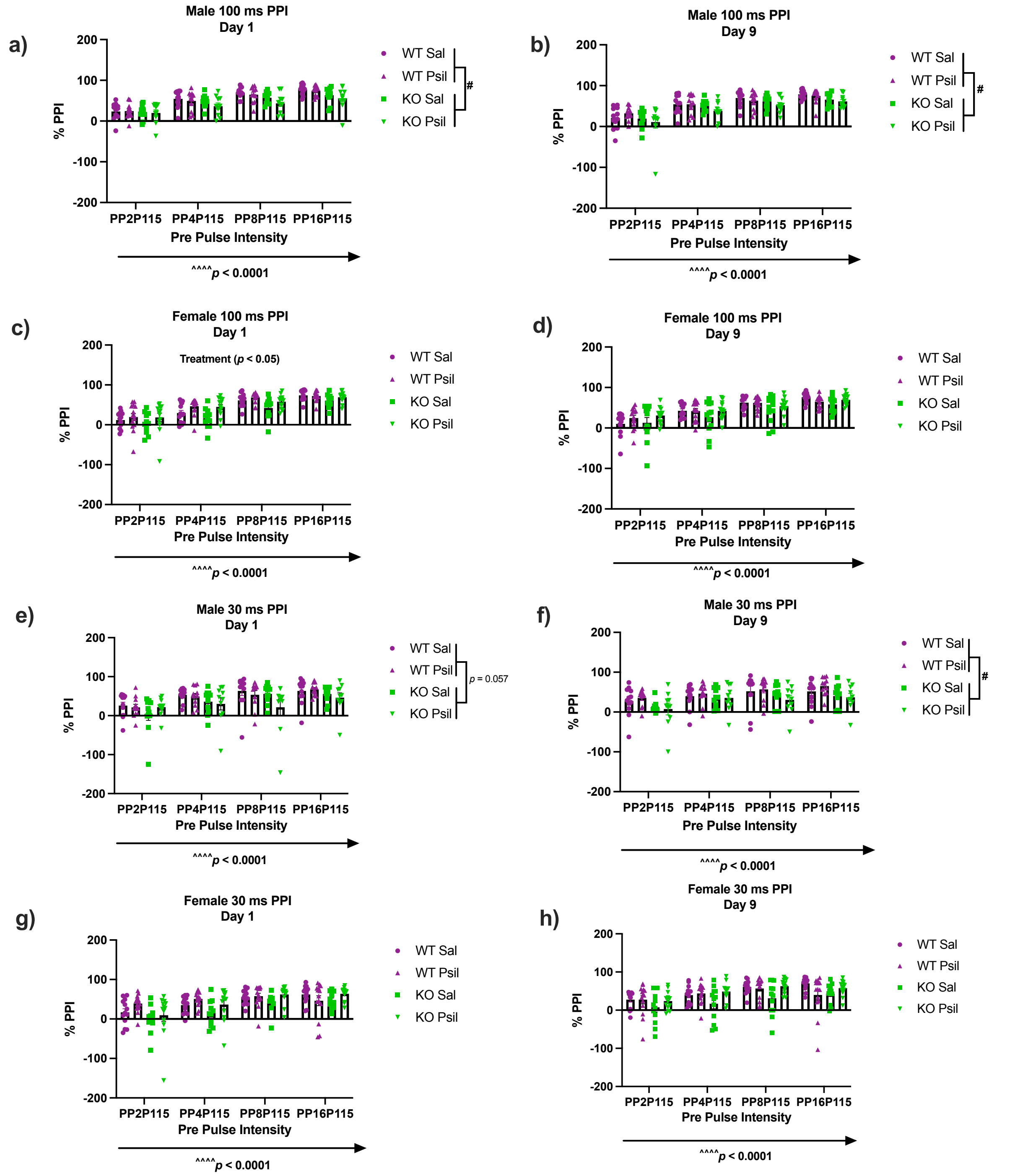

Supplementary Figure 9. Pre-pulse inhibition by intensity

a-h) Across all figure panels, PPI increased as intensity increased ($p < 0.0001$). **a)** When looking at 100 ms PPI across the varying prepulse intensities in male mice 1 day after injection, that KO mice had reduced PPI compared to WT mice ($p = 0.024$). There was no main effect of treatment ($p = 0.225$) or any significant interaction. **b)** At day 9, there was a similar main effect of genotype ($p = 0.040$), and no effect of treatment ($p = 0.448$) or any significant interactions. **c)** For females (100 ms PPI) at day 1 post injection, there was a main effect of treatment ($p = 0.018$), with psilocybin significantly increasing %PPI compared to saline. There was no significant genotype effect ($p = 0.083$) or any significant interactions. **d)** At day 9 post injection (100 ms PPI), we found a significant intensity × genotype interaction ($p = 0.046$),

however post hoc analysis revealed no significant group level difference ($p \geq 0.212$). There were no other significant effects. **e)** For male day 1 (30 ms PPI across intensities), there was no significant main effect of treatment ($p = 0.497$) or genotype ($p = 0.057$). There were no significant interactions. **f)** For male day 9 (30 ms PPI across intensities) there was a main effect of genotype ($p = 0.037$) but not treatment ($p = 0.816$) or any significant interaction. **g)** When analyzing female day 1, 30 ms PPI across varying prepulse intensities, we found a significant genotype × intensity interaction ($p = 0.030$), but post hoc analysis found no group level difference ($p \geq 0.143$) and there were no other significant effects. **h)** For day 9 (30 ms PPI across intensities in females), we found a genotype × treatment interaction ($p = 0.040$), however, post hoc analysis did not find any significant effects ($p \geq 0.099$). Likewise, an intensity × treatment effect was observed ($p = 0.009$) but again, post hoc results were non-significant ($p \geq 0.183$). Bars represent mean ± SEM; individual data points represent mice. WT = wild-type, KO = knockout, Sal = saline, Psil = psilocybin 1 mg/kg. $^{\wedge\wedge\wedge\wedge}p < 0.0001$ main effect of prepulse intensity. $^{\#}p < 0.05$, WT vs KO. For panel c, the bold text above the grouped bars represents a significant main effect of treatment.

References

1. Gattuso JJ, Wilson C, Hannan AJ, Renoir T. Acute administration of the NMDA receptor antagonists ketamine and MK-801 reveals dysregulation of glutamatergic signalling and sensorimotor gating in the Sapap3 knockout mouse model of compulsive-like behaviour. Neuropharmacology. 2023;239:109689.
2. Burrows EL, McOmish CE, Buret LS, Van den Buuse M, Hannan AJ. Environmental enrichment ameliorates behavioral impairments modeling schizophrenia in mice lacking metabotropic glutamate receptor 5. Neuropsychopharmacology. 2015;40:1947–1956.
3. Valsamis B, Schmid S. Habituation and prepulse inhibition of acoustic startle in rodents. J Vis Exp. 2011:3446.
4. Paylor R, Crawley JN. Inbred strain differences in prepulse inhibition of the mouse startle response. Psychopharmacology (Berl). 1997;132:169–180.
5. Brody SA, Conquet F, Geyer MA. Effect of antipsychotic treatment on the prepulse inhibition deficit of mGluR5 knockout mice. Psychopharmacology (Berl). 2004;172:187–195.
6. Payet JM, Burnie E, Sathananthan NJ, Russo AM, Lawther AJ, Kent S, et al. Exposure to acute and chronic fluoxetine has differential effects on sociability and activity of serotonergic neurons in the dorsal raphe nucleus of juvenile male BALB/c mice. Neuroscience. 2018;386:1–15.
7. Willins DL, Meltzer HY. Direct injection of 5-HT2A receptor agonists into the medial prefrontal cortex produces a head-twitch response in rats. J Pharmacol Exp Ther. 1997;282:699–706.
8. Shao L-X, Liao C, Gregg I, Davoudian PA, Savalia NK, Delagarza K, et al. Psilocybin induces rapid and persistent growth of dendritic spines in frontal cortex in vivo. Neuron. 2021;109:2535–2544.
9. Pomarol-Clotet E, Canales-Rodríguez EJ, Salvador R, Sarro S, Gomar JJ, Vila F, et al. Medial prefrontal cortex pathology in schizophrenia as revealed by convergent findings from multimodal imaging. Mol Psychiatry. 2010;15:823–830.
10. Barbera G, Liang B, Zhang L, Gerfen CR, Culurciello E, Chen R, et al. Spatially compact neural clusters in the dorsal striatum encode locomotion relevant information. Neuron. 2016;92:202–213.
11. Durieux PF, Schiffmann SN, de Kerchove d'Exaerde A. Differential regulation of motor control and response to dopaminergic drugs by D1R and D2R neurons in distinct dorsal striatum subregions. EMBO J. 2011;31:640.
12. Révy D, Jaouen F, Salin P, Melon C, Chabbert D, Tafi E, et al. Cellular and behavioral outcomes of dorsal striatonigral neuron ablation: new insights into striatal functions. Neuropsychopharmacology. 2014;39:2662–2672.
13. Hall H, Farde L, Halldin C, Lundkvist C, Sedvall G. Autoradiographic localization of 5-HT2A receptors in the human brain using [3H] M100907 and [11C] M100907. Synapse. 2000;38:421–431.
14. Pompeiano M, Palacios JM, Mengod G. Distribution of the serotonin 5-HT2 receptor family mRNAs: comparison between 5-HT2A and 5-HT2C receptors. Molecular Brain Research. 1994;23:163–178.
15. Barrett FS, Krimmel SR, Griffiths RR, Seminowicz DA, Mathur BN. Psilocybin acutely alters the functional connectivity of the claustrum with brain networks that support perception, memory, and attention. Neuroimage. 2020;218:116980.
16. Cascella NG, Sawa A. The claustrum in schizophrenia. The claustrum, Elsevier; 2014. p. 237–243.

17. Bates D. lme4: Linear mixed-effects models using Eigen and S4. R Package Version. 2016;1:1.
18. Kuznetsova A, Brockhoff PB, Christensen RHB. lmerTest Package: Tests in Linear Mixed Effects Models. J Stat Softw. 2017;82:1–26.
19. Lenth R. emmeans: estimated marginal means, aka least-squares means. R package version 1.8.7. 2023.
20. Frossard J, Renaud O. Permutation tests for regression, ANOVA, and comparison of signals: the permuco package. J Stat Softw. 2021;99:1–32.
21. Villacorta PJ. The welchADF Package for Robust Hypothesis Testing in Unbalanced Multivariate Mixed Models with Heteroscedastic and Non-normal Data. R J. 2017;9.
22. Wickham H. ggplot2. Wiley Interdiscip Rev Comput Stat. 2011;3:180–185.
